# Artificial Intelligence for Predicting Treatment Response in Patients With Anxiety Disorders After Cognitive Behavioral Therapy: Systematic Review and Meta-Analysis

**DOI:** 10.2196/86079

**Published:** 2026-03-18

**Authors:** Jiawen Liu, Junhui Wang, Zhaobin Wu, Mohamad Ibrani Shahrimin Bin Adam Assim

**Affiliations:** 1 Youth League Committee, Liuzhou Railway Vocational Technical College, No. 2 Wenyuan Road, Yufeng District, Liuzhou, Guangxi Zhuang Autonomous Region, 545000, China, 60 1116670058; 2 Faculty of Humanities, Management and Science, Universiti Putra Malaysia, Bintulu, Sarawak, Malaysia; 3 School of Automation, Guangxi University of Science and Technology, Liuzhou, Guangxi, China

**Keywords:** artificial intelligence, treatment response, anxiety disorders, cognitive behavioral therapy, meta-analysis

## Abstract

**Background:**

Artificial intelligence (AI) models have been increasingly explored for predicting treatment response to cognitive behavioral therapy (CBT) in patients with anxiety disorders. Identifying potential responders in advance may help inform treatment planning and support clinical decision-making. Although a growing number of studies have applied AI techniques in this context, reported performance estimates vary across studies, and the overall predictive accuracy has not been comprehensively quantified.

**Objective:**

This systematic review and meta-analysis aims to quantify the overall performance of AI models in predicting treatment response following CBT for anxiety disorders and to examine how data sources, algorithmic approaches, and diagnostic subtypes influence predictive performance.

**Methods:**

A systematic literature search was conducted in PubMed, Embase, Web of Science, Cochrane Library, and PsycINFO up to August 2025. We included studies that validated AI models for predicting CBT treatment response (remission or response) in patients diagnosed with an anxiety disorder. The risk of bias was assessed using the PROBAST+AI (Prediction Model Risk of Bias Assessment Tool for Artificial Intelligence) tool. Predictive performance metrics, including sensitivity, specificity, accuracy, and area under the curve (AUC), were extracted and pooled. Pooled estimates for sensitivity, specificity, and diagnostic accuracy were derived using the Restricted Maximum Likelihood estimator, with CIs adjusted via the Hartung-Knapp-Sidik-Jonkman method. Prediction intervals were calculated and reported alongside these pooled estimates to illustrate the expected distribution of effects in real-world settings.

**Results:**

Eleven studies were included in the meta-analysis. The pooled sensitivity of AI-based models for predicting treatment response was 0.73 (95% CI 0.58‐0.85; *I*²=82.8%), and the pooled specificity was 0.75 (95% CI 0.59‐0.89; *I*²=96.7%). The overall pooled accuracy was 0.74 (95% CI 0.62‐0.84; *I*²=94.6%). The summary AUC was 0.81 (95% CI 0.78‐0.85), indicating moderate discriminative performance. Subgroup analyses showed that models incorporating multimodal data achieved superior predictive performance, with a pooled sensitivity of 0.84 and an accuracy of 0.82. In addition, predictive performance was the highest in patients with social anxiety disorder compared with other anxiety disorder subtypes.

**Conclusions:**

This meta-analysis quantitatively synthesized AI performance in predicting CBT response for anxiety disorders, moving beyond narrative reviews to provide pooled evidence. In contrast to existing reviews that encompass broader diagnostic groups, our focused approach establishes a precise benchmark for this clinical domain, highlighting the current moderate overall performance. Furthermore, we extend beyond previous work by demonstrating the superior predictive utility of multimodal data, identifying social anxiety disorder as the most predictable subtype, and systematically evaluating the impact of data modalities and algorithm types. Future efforts should prioritize robustly validated multimodal models, laying essential groundwork for the potential development of AI-assisted tools to personalize treatment planning in anxiety disorders.

## Introduction

Anxiety disorders constitute a group of mental disorders characterized by intense, excessive, and persistent worry and fear, exhibiting high prevalence rates. They not only cause significant functional impairment in patients’ social, academic, and occupational functioning but also impose substantial socioeconomic burdens at the societal level, including considerable health care resource consumption and productivity losses [1,2]. Cognitive behavioral therapy (CBT) is a first-line evidence-based intervention for anxiety disorders [3,4]. However, treatment response varies considerably, with a notable subset of patients showing suboptimal or inadequate improvement [5]. This heterogeneity underscores a critical clinical need: the ability to preemptively identify likely responders and, more importantly, those at high risk of nonresponse who may benefit from treatment augmentation or alternative interventions from the outset [6]. Early and accurate prediction of treatment response can optimize care pathways, improve resource allocation, and reduce delays, aligning with the goals of precision mental health [7].

Clinical assessment of anxiety disorders typically relies on structured or semistructured interviews and standardized scales [8], such as the Hamilton Anxiety Rating Scale (HAM-A) [9], Clinical Global Impressions–Improvement (CGI-I) [10], and Liebowitz Social Anxiety Scale (LSAS) [7], which are fundamental for symptom identification, outcome evaluation, and longitudinal monitoring. However, they possess inherent limitations for predictive purposes. First, they depend heavily on self-report and subjective ratings, making them vulnerable to recall bias and social desirability effects, thereby compromising objectivity [11]. Second, heterogeneity in measured constructs, scoring procedures, and cutoff thresholds across instruments constrains comparability and diagnostic consistency across studies and clinical settings [12]. Consequently, there is growing recognition that these traditional metrics alone may be insufficient for reliably forecasting an individual’s unique response trajectory to a specific therapy such as CBT [13].

Against this background, artificial intelligence (AI), particularly machine learning (ML) and deep learning (DL), has attracted increasing attention as a methodological framework for advancing outcome prediction in mental health research. AI-based models can integrate large-scale, high-dimensional, and multimodal data and capture complex, nonlinear relationships that are difficult to model with conventional statistical techniques [14]. In recent years, a growing number of studies have applied ML and DL methods to predict treatment response across a range of psychiatric interventions, including CBT for anxiety disorders [15,16]. These studies have incorporated diverse data sources, including baseline clinical characteristics, symptom trajectories, neuroimaging markers, psychophysiological measures, and digital behavioral indicators, and have reported varying levels of predictive accuracy [17,18]. However, the anxiety disorders comprise clinically distinct subtypes, such as social anxiety disorder (SAD), generalized anxiety disorder (GAD), and panic disorder (PD), which differ in symptom profiles and underlying mechanisms [19]. This diagnostic heterogeneity may further influence model generalizability and predictive stability across populations [7,20]. To date, however, quantitative evidence synthesizing the predictive performance of AI models for CBT response in different anxiety disorder subtypes remains limited, and potential sources of heterogeneity across studies remain insufficiently explored and require systematic investigation [21].

This systematic review and meta-analysis aims to quantitatively synthesize the overall performance of AI models in predicting treatment response following CBT for anxiety disorders. We also seek to explore the influence of key factors, such as data sources, algorithmic approaches, and diagnostic subtypes, on predictive performance and to preliminarily investigate potential sources of the observed heterogeneity.

## Methods

### Ethical Considerations

As this is a systematic review and meta-analysis, ethics approval and consent to participate are not applicable. The manuscript does not include the identification images or other personal or clinical details of participants.

The meta-analysis adhered rigorously to the PRISMA-DTA (Preferred Reporting Items for Systematic Reviews and Meta-Analyses of Diagnostic Test Accuracy) reporting guidelines [22] and the PRISMA-S extension for reporting literature searches [23]. We have provided the completed PRISMA-DTA (Checklist 1), PRISMA 2020 Abstract (Checklist 2), and PRISMA-S (Checklist 3) checklists. The research protocol was registered in the PROSPERO registry (registration ID: CRD420251137096).

### Feasibility Assessment for Meta-Analysis

Prior to initiating the full systematic review, a preliminary scoping search was conducted to assess the feasibility of quantitative synthesis. This step aimed to determine whether sufficient studies reported the extractable predictive performance metrics necessary for a meta-analysis. In the scoping search, although the field is nascent, several published studies met the core data requirements, supporting the rationale for proceeding with a formal systematic review and meta-analysis.

### Search Strategy

A systematic search was conducted in PubMed, Embase, Web of Science, Cochrane Library, and PsycINFO from inception to August 16, 2025. Two independent reviewers (JL and JW) screened titles/abstracts and subsequently assessed full texts, with disagreements resolved by consensus. Search strategies combined controlled vocabulary (MeSH) and free-text terms across 3 concept blocks: AI and methods (eg, “artificial intelligence,” “machine learning,” and “deep learning”), anxiety disorders (eg, “anxiety disorders,” “social anxiety disorder,” “generalized anxiety disorder,” and “panic disorder”), and CBT (eg, “cognitive behavioral therapy,” “CBT,” and “cognitive therapy”). No language or publication year limits were applied at the initial search stage to minimize selection bias. Detailed database-specific strategies are provided in Table S1 in Multimedia Appendix 1. Study selection followed a dual-stage independent screening process. Two reviewers (JL and JW) first screened titles/abstracts and then independently assessed full texts of potentially eligible records. Disagreements were resolved by consensus or by third-party arbitration (ZW). The reference lists of included studies were manually screened for additional records. It should be noted that in conducting this systematic review, we did not contact authors, experts, or manufacturers to obtain additional studies or data, and did not adapt or reuse search strategies from previous reviews. Additionally, a formal peer review of the search strategy was not conducted.

### Inclusion and Exclusion Criteria

The PITROS framework was used for the inclusion criteria:

- Participants (P): patients with anxiety disorders receiving CBT, including separation anxiety disorder, selective mutism, specific phobia, SAD, PD/panic attacks, agoraphobia, GAD, and substance/medication-induced anxiety disorder
- Index test (I): AI methods (eg, ML and DL) developed to predict CBT treatment response
- Target condition (T): positive cases defined as clinically significant post-CBT improvement (eg, meaningful symptom reduction); negative cases defined as poor or no response
- Reference standard (R): validated diagnostic/severity scales for anxiety disorders
- Outcomes (O): sensitivity, specificity, area under the curve (AUC), and accuracy
- Setting (S): mental health outpatient clinics, psychiatric hospitals, and clinical trial centers; multicenter prospective or retrospective designs using electronic health records or public databases

The exclusion criteria were as follows: (1) publication in a language other than English at the full-text stage, (2) nonempirical article type (eg, review, case report, conference abstract, meta-analysis, and letter), (3) no assessment of the prediction of CBT response using AI, (4) inclusion of a population that did not have anxiety disorder or that did not undergo CBT, (5) absence of a recognized reference standard or inadequate data to derive the target metrics, and (6) duplicate publication identified using EndNote and manual checking. The database search was unrestricted by language to maximize sensitivity, but non-English publications were excluded during full-text review owing to translation resource constraints. We acknowledge this as a potential limitation, as it may have introduced language bias by excluding relevant studies published in other languages. The independent selection of studies was conducted by 2 reviewers (JL and JW), with disagreements resolved through discussion or by a third reviewer (ZW).

### Data Extraction

Two independent reviewers (JL and JW) conducted comprehensive data extraction from full-text articles to assess study eligibility. The extracted data encompassed critical study characteristics, including the authors, year, country, study design, center, response definition, types of disorders, and types of treatments. Specific quantitative elements included the total number of patients in the training and internal validation sets, the number of response patients in the training and internal validation sets, the data-splitting method, predictors, AI algorithms, and AI methods. For internal validation sets, we systematically recorded true positive (TP), true negative (TN), false positive (FP), and false negative (FN) values.

For studies included in the systematic review but lacking meta-analysis–compatible data, we proactively contacted corresponding authors via email to request necessary information. Given the frequent absence of diagnostic contingency tables, we reconstructed 2×2 tables using reported sensitivity, specificity, responder numbers, and total patient counts. Any discrepancies in extraction were resolved through a collaborative discussion, with a third reviewer (ZW) serving as an arbitrator if consensus could not be reached, thereby ensuring methodological rigor and data integrity.

### Quality Assessment

The latest PROBAST+AI (Prediction Model Risk of Bias Assessment Tool for Artificial Intelligence) quality assessment tool was used [24], which replaced PROBAST 2019. This tool comprises 2 phases: model development and model evaluation. Each phase encompasses 7 domains covering participant and data sources, predictors, outcome assessment, and analysis. Evaluation results for each domain are categorized as “low,” “high,” or “unclear,” determined based on specific signal questions. Signal concerns are rated as “yes,” “probably yes,” “probably no,” “no,” “no information,” and, in some cases, “not applicable.” Signal concerns phrased as “yes” or “probably yes” indicate a lower risk of bias. Any signal question rated “no” or “probably no” indicates a potential high risk of bias in that domain. If no “no” or “probably no” rating exists but “no information” is present, the domain’s bias risk is classified as unclear. The complete list of signal questions and tables is detailed in Tables S2 and S3 in Multimedia Appendix 1.

To ensure objectivity and accuracy in the assessment process, 2 reviewers (JL and JW) independently assessed the risk of bias in the included studies using the PROBAST+AI quality assessment tool. During the review process, any discrepancies between reviewers were resolved through in-depth discussions and analyses to achieve consensus, ensuring highly reliable, consistent final assessment results.

### Outcome Measures

The primary outcome measures encompassed comprehensive diagnostic performance metrics from internal validation sets, including sensitivity, specificity, accuracy, and AUC. Sensitivity, calculated as TP/(TP+FN), measured the AI model’s ability to accurately identify TPs, while specificity, computed as TN/(TN+FP), assessed the model’s proficiency in correctly identifying negative cases. AUC provided a holistic assessment of the model’s discriminative power between positive and negative instances. Accuracy, defined as (TP+TN)/total patients, represented the proportion of correctly classified samples. We systematically extracted performance metrics from all data sources (eg, imaging, clinical/demographic, and multimodal) and AI algorithms presented in each study. However, for the purpose of meta-analytic pooling, only the primary model from each article, specifically the model based on nonoverlapping patient cohorts, was selected to ensure data independence and avoid double-counting.

### Statistical Analysis

While the core analytical plan and reporting adhered to the preregistered protocol, a key methodological deviation was implemented to enhance the robustness of our statistical synthesis. The registered protocol specified the use of the bivariate random-effects model (Reitsma model) as the primary method for pooling sensitivity and specificity. In the final analysis, we maintained this model for generating the summary receiver operating characteristic (SROC) curve and the AUC. However, for the pooled estimates of sensitivity and specificity themselves, we adopted a univariate random-effects model using the Restricted Maximum Likelihood estimator, with CIs adjusted via the Hartung-Knapp-Sidik-Jonkman (HKSJ) method. This adaptation was made to incorporate the HKSJ adjustment, which provides more conservative and reliable CIs, particularly when dealing with a limited number of studies or substantial heterogeneity [25]. Prediction intervals (PIs) were calculated and reported alongside these pooled estimates to illustrate the expected distribution of effects in real-world settings. Heterogeneity was quantitatively assessed using the PIs and further explored through subgroup analyses and meta-regression [26]. Bivariate boxplots were used to visually identify potential outlier studies contributing to heterogeneity. Prespecified covariates for meta-regression included the number of study centers, anxiety disorder subtype, data splitting method, CBT protocol type, and AI algorithm category. Subgroup analyses were conducted according to data type, algorithm type, and specific anxiety disorder diagnoses. Additionally, a bubble plot was used to assess temporal trends in the accuracy of different algorithms, and funnel plots were used, along with Egger tests, to evaluate potential small-study effects.

## Results

### Study Selection

**Figure 1.**
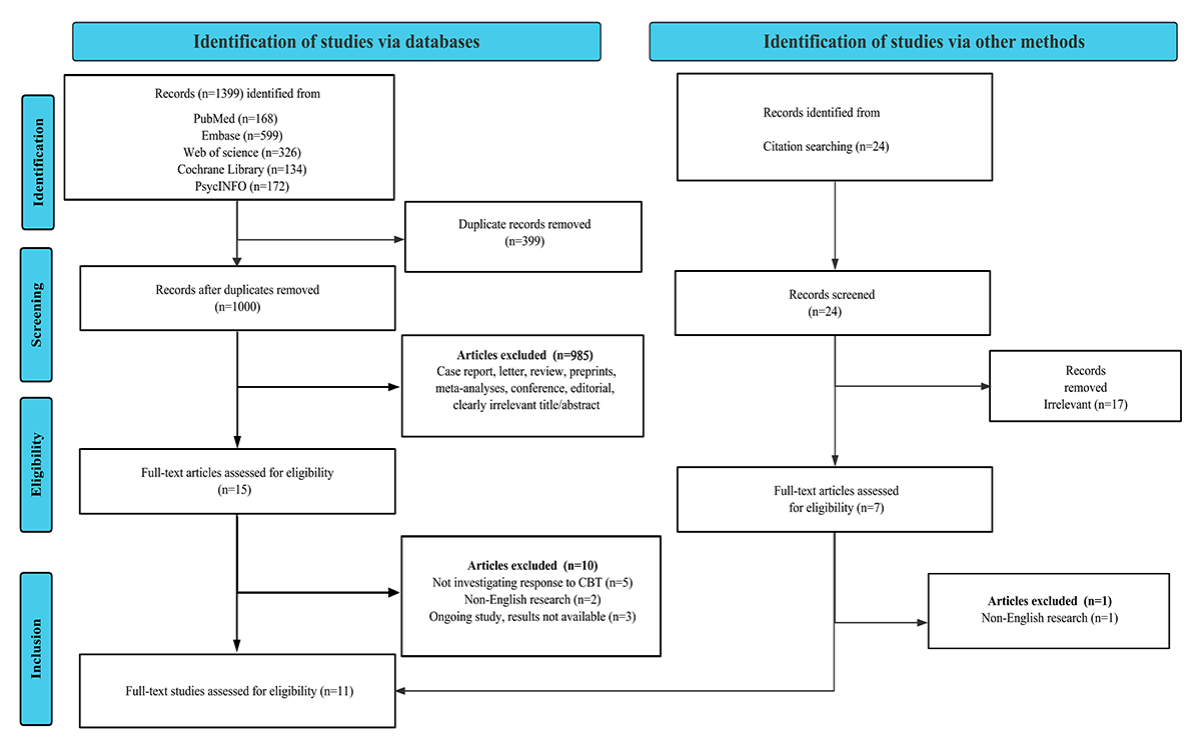
PRISMA (Preferred Reporting Items for Systematic Reviews and Meta-Analyses) s in Figure 1: flow diagram of the systematic literature search and study selection process for studies evaluating artificial intelligence models in predicting treatment response to cognitive behavioral therapy for anxiety disorders.

A search across 5 electronic databases yielded 1399 potentially relevant publications. After deleting 399 duplicates, 1000 records were retained for preliminary screening. At this phase, 980 studies were identified as clearly irrelevant based on the title/abstract and the publication type. Thus, 15 articles were selected for full-text review. Following a comprehensive assessment of the full texts, 5 studies were removed because they did not investigate treatment response to CBT. Two studies were excluded due to non-English publication. Three studies were excluded as they were ongoing trials without any published outcome measures. Additionally, 6 qualifying studies were identified from nondatabase sources. Ultimately, 11 studies [7,17,18,27-34] were included in the meta-analysis as they met predefined inclusion criteria. The identification, screening, eligibility assessment, and inclusion process followed the PRISMA guidelines, and the selection analysis is elaborated in Figure 1.

### Study Description and Quality Assessment

A total of 11 eligible studies comprising 59,085 samples (training: 38,563; internal validation set: 20,522) were included. Among these, 4 studies exclusively evaluated SAD [7,29,32,34], 2 studies focused on PD with agoraphobia [30,33], 1 study investigated both GAD and PD [27], 1 study examined PD and SAD [31], and the remaining 3 studies addressed multiple anxiety disorder subtypes [17,18,28]. Nine studies [17,18,27,29-34] provided data eligible for meta-analysis. The included studies were published between 2014 and 2024, and all were prospective. Definitions of treatment response varied across studies: 2 studies used the CGI-I [29,32], 2 used the LSAS [7,34], 2 used the HAM-A [30,33], 1 used the Overall Anxiety Severity and Impairment Scale (OASIS) [27], 1 used the clinical severity rating [28], 1 combined HAM-A and the Spider Phobia Questionnaire (SPQ) [18], 1 used both the Generalized Anxiety Disorder-7 (GAD-7) and the Patient Health Questionnaire-9 (PHQ-9) [17], and 1 applied multiple scales simultaneously [31]. Across the 9 datasets, the most frequently used AI algorithms were support vector machines (SVMs; 4/9, 44%) and random forests (RFs; 3/9, 33%). Additionally, of the 9 datasets, 2 were based on clinical/demographic data, 6 on imaging data, and 1 on multimodal data. The clinical, methodological, and technical characteristics of the included studies are summarized in Table 1 and Multimedia Appendix 2.

**Table 1.** Characteristics of the included studies (n=11) and their patient populations.

Study (author)	Year	Country	Study design	Center	Response definition	Types of anxiety disorders	Types of treatments	Data source	Total sample size, n		Response sample size, n	
									Training	Internal validation	Training	Internal validation
Ball et al [27]	2014	America	Pro^a^	Single center	OASIS^b^ ≤5	GAD^c^, PD^d^	Standard CBT^e^	Institutional database	48	49	29	49
Bertie et al [28]	2024	Multiple countries	Pro	Multiple centers	CSR^f^ ≥4	GAD, separation anxiety, SAD^g^, SPH^h^	Standard CBT	Institutional database	2214	2214	—^i^	—
Bukhari et al [7]	2025	America	Pro	Multiple centers	LSAS^j^↓ ≥50%	SAD	Standard CBT	Institutional database	157	157	—	—
Frick et al [29]	2020	Sweden	Pro	Single center	CGI-I^k^ ≤2	SAD	CBT+SSRI^l^, CBT+placebo	Institutional database	47	47	24	24
Hahn et al [30]	2015	Germany	Pro	Multiple centers	HAM-A^m^↓ ≥50%	PD with AG^n^	Standard CBT	Institutional database	49	49	25	25
Hilbert et al [18]	2024	Germany	Pro	Multiple centers	Protect-AD: HAM-A↓ ≥50%; Spider VR: SPQ^o^↓ ≥30%	PD, AG, SAD, SPH	Standard CBT	Institutional database	410	410	213	213
Hentati Isacsson et al [31]	2024	Sweden	Pro	Single center	MADRS^p^ ≤11, PDSS^q^ ≤8, LSAS ≤35	PD, SAD	iCBT^r^	Institutional database	3619	3619	—	—
Månsson et al [32]	2015	Sweden	Pro	Multiple centers	CGI-I ≤2	SAD	iCBT	Institutional database	23	23	12	12
Prasad et al [17]	2023	United Kingdom, America	Pro	Multiple centers	GAD-7^s^↓ ≥64, PHQ-9^t^↑ <6	Anxiety	iCBT	Institutional database	31,899	13,857	—	5232
Sundermann et al [33]	2017	Germany	Pro	Multiple centers	HAM-A↓ ≥50%	PD with AG	Standard CBT	Institutional database	59	59	30	30
Whitfield-Gabrieli et al [34]	2015	America	Pro	Multiple centers	LSAS↓ ≥50%	SAD	Standard CBT	Institutional database	38	38	19	19

a Pro: prospective.

b OASIS: Overall Anxiety Severity and Impairment Scale.

c GAD: generalized anxiety disorder.

d PD: panic disorder.

e CBT: cognitive behavioral therapy.

f CSR: clinical severity rating.

g SAD: social anxiety disorder.

h SPH: specific phobia.

i Not applicable.

j LASA: Liebowitz Social Anxiety Scale.

k CGI-I: clinical global impression-improvement.

l SSRI: selective serotonin reuptake inhibitor.

m HAM-A: Hamilton Anxiety Rating Scale.

n AG: agoraphobia.

o SPQ: Spider Phobia Questionnaire.

p MADRS: Montgomery-Asberg Depression Rating Scale.

q PDSS: Panic Disorder Severity Scale.

r iCBT: internet-based cognitive behavioral therapy.

s GAD-7: Generalized Anxiety Disorder-7.

t PHQ-9: Patient Health Questionnaire-9.

**Figure 2.**
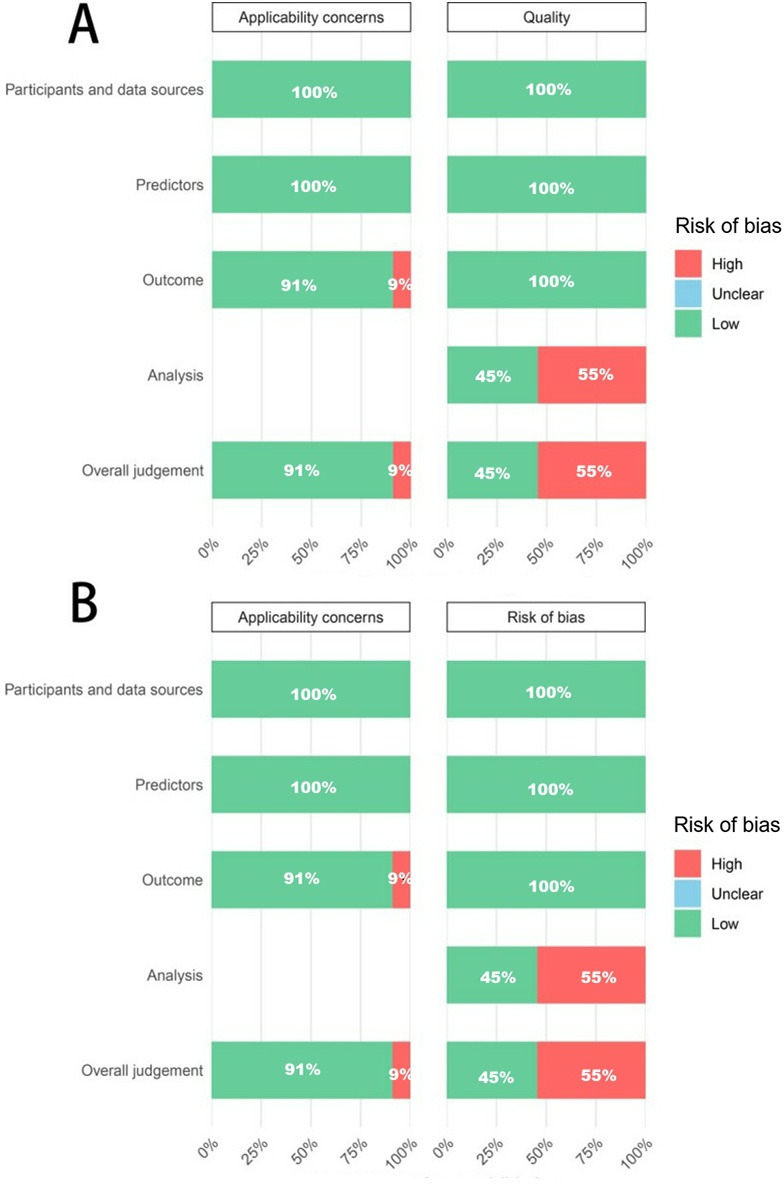
Risk of bias and applicability assessment using the PROBAST+AI tool for studies developing or validating AI models to predict treatment response after cognitive behavioral therapy in patients with anxiety disorders. Panel A summarizes the risk of bias for model development studies, and panel B summarizes the risk of bias for model validation studies. PROBAST+AI: Prediction Model Risk of Bias Assessment Tool for Artificial Intelligence.

The risk of bias, as assessed by the PROBAST+AI quality assessment tool, is presented in Figure 2 and Tables S2 and S3 in Multimedia Appendix 1. For model development, in the overall quality assessment, 55% (6/11) of studies were rated as high risk, while the remaining 45% (5/11) were rated as low risk. Regarding applicability concerns, 9% (1/11) of studies were rated as high risk, while the remaining 91% (10/11) were rated as low risk. For model validation, 55% (6/11) of studies were rated as high risk in the overall risk of bias assessment, with the remaining 45% (5/11) rated as low risk. In the comprehensive evaluation of applicability concerns, 9% (1/11) of studies were rated as high risk, while the remaining 91% (10/11) were rated as low risk.

### Trend in Diagnostic Accuracy Over Time

**Figure 3.**
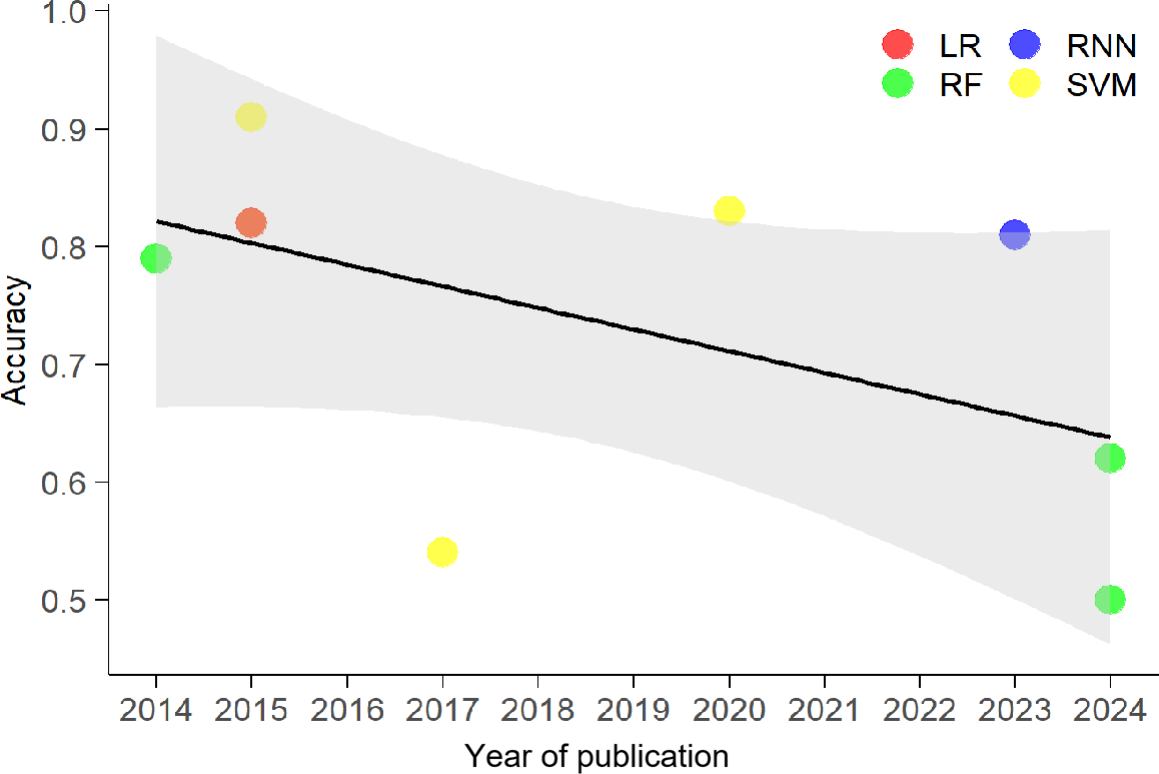
Bubble plot illustrating the accuracy of various artificial intelligence algorithms over time (publication year) for predicting treatment response to cognitive behavioral therapy in patients with anxiety disorders. LR: logistic regression; RF: random forest; RNN: recurrent neural network; SVM: support vector machine.

The scatter plot in Figure 3 illustrates a negative trend in model accuracy from 2014 to 2024 across logistic regression (LR), recurrent neural network (RNN), RF, and SVM algorithms. A linear regression line confirms that reported accuracy has generally decreased over the decade within this dataset. The highest performance was recorded in 2015 by an SVM model (approximately 0.9), while the lowest scores appeared in 2024, with RF models showing a drop in scores to around 0.5. The shaded region indicates the CI for this downward trajectory (Figure 3).

### Predicting CBT Response to Anxiety Disorders Using AI Models Based on the Primary Model Per Study

**Figure 4.**
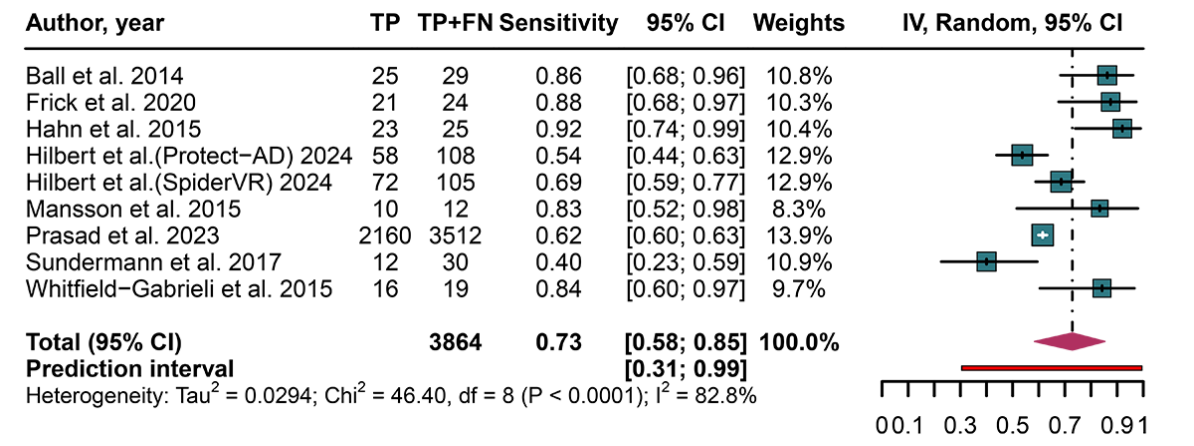
Forest plot displaying the pooled sensitivity, with 95% CIs and prediction intervals, of artificial intelligence models for predicting treatment response after cognitive behavioral therapy in patients with anxiety disorders [17,18,27,29,30,32-34]. FN: false negative; TP: true positive.

**Figure 5.**
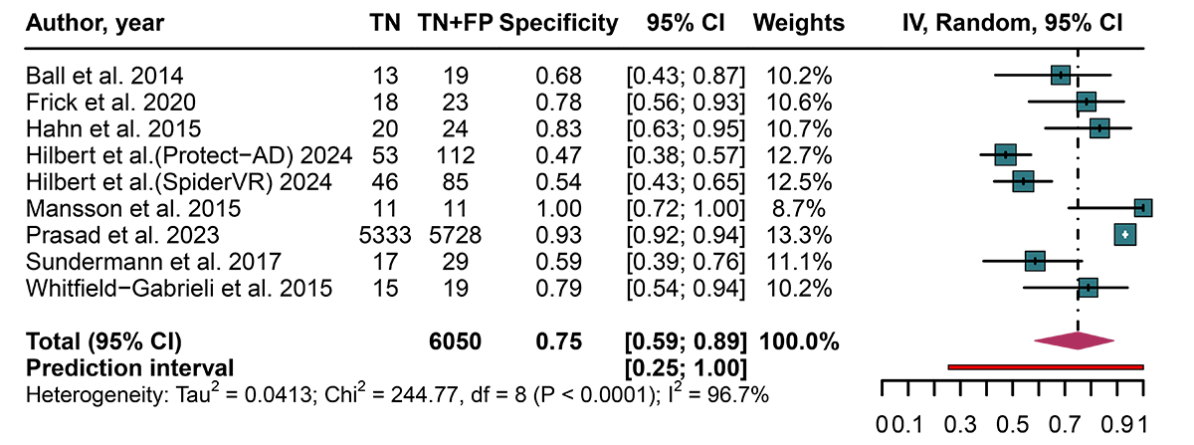
Forest plot displaying the pooled specificity, with 95% CIs and prediction intervals, of artificial intelligence models for predicting treatment response after cognitive behavioral therapy in patients with anxiety disorders [17,18,27,29,30,32-34]. FP: false positive; TN: true negative.

**Figure 6.**
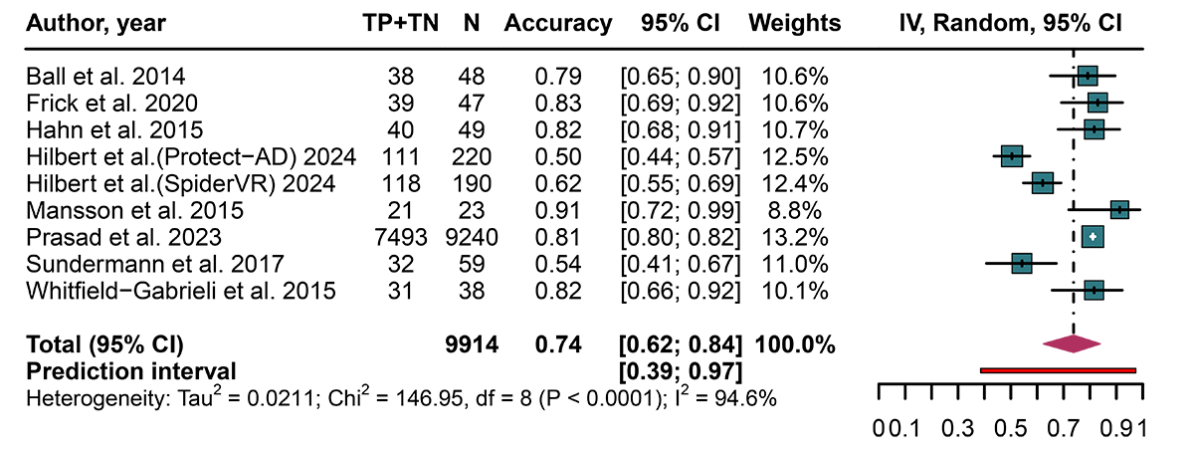
Forest plot displaying the pooled accuracy, with 95% CIs and prediction intervals, of artificial intelligence models for predicting treatment response after cognitive behavioral therapy in patients with anxiety disorders [17,18,27,29,30,32-34]. TN: true negative; TP: true positive.

**Figure 7.**
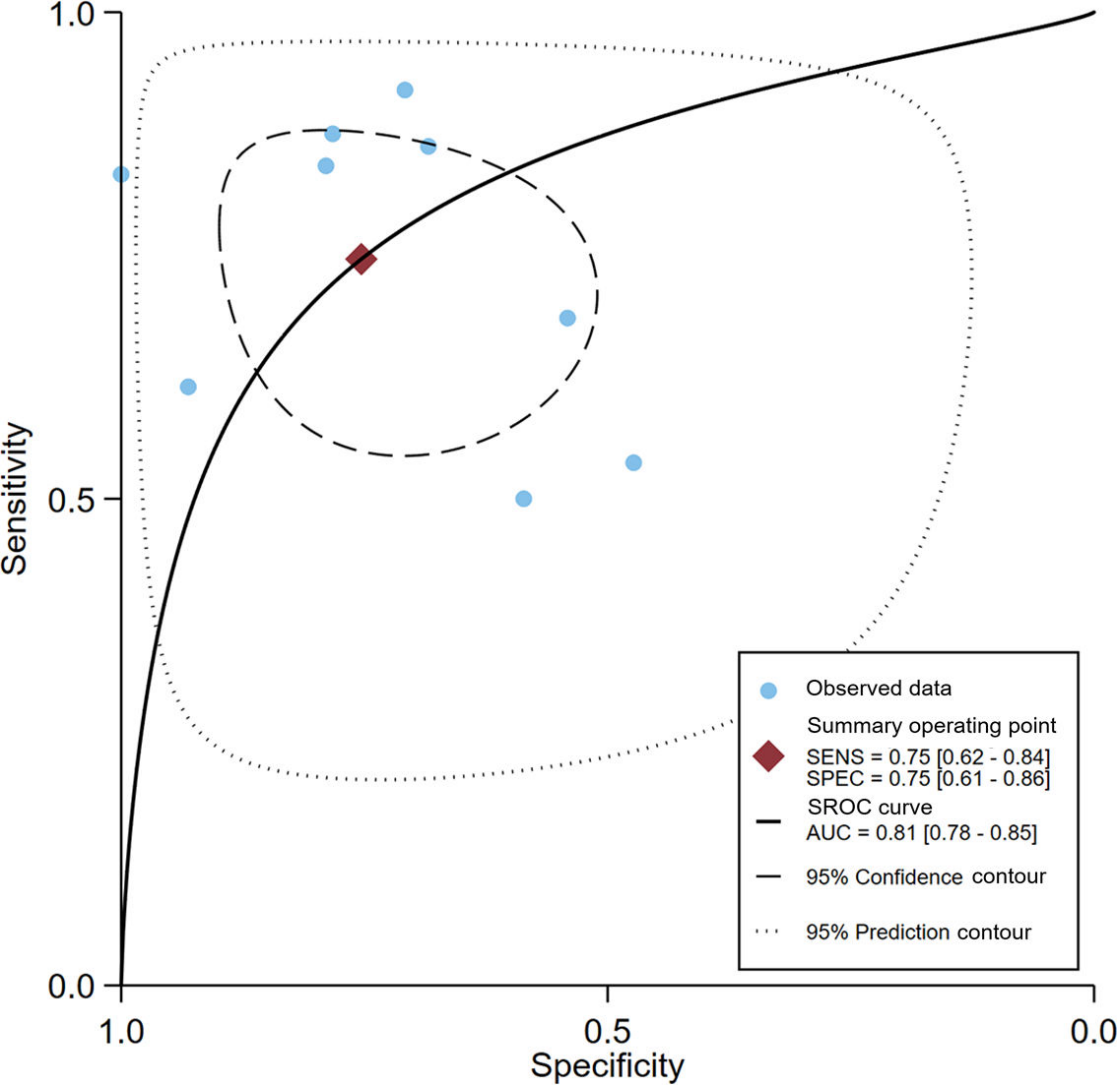
Summary receiver operating characteristic (SROC) curve for artificial intelligence models predicting treatment response in patients with anxiety disorders after cognitive behavioral therapy. The summary point estimates for sensitivity (SENS) and specificity (SPEC), and the area under the curve (AUC) are shown.

**Figure 8.**
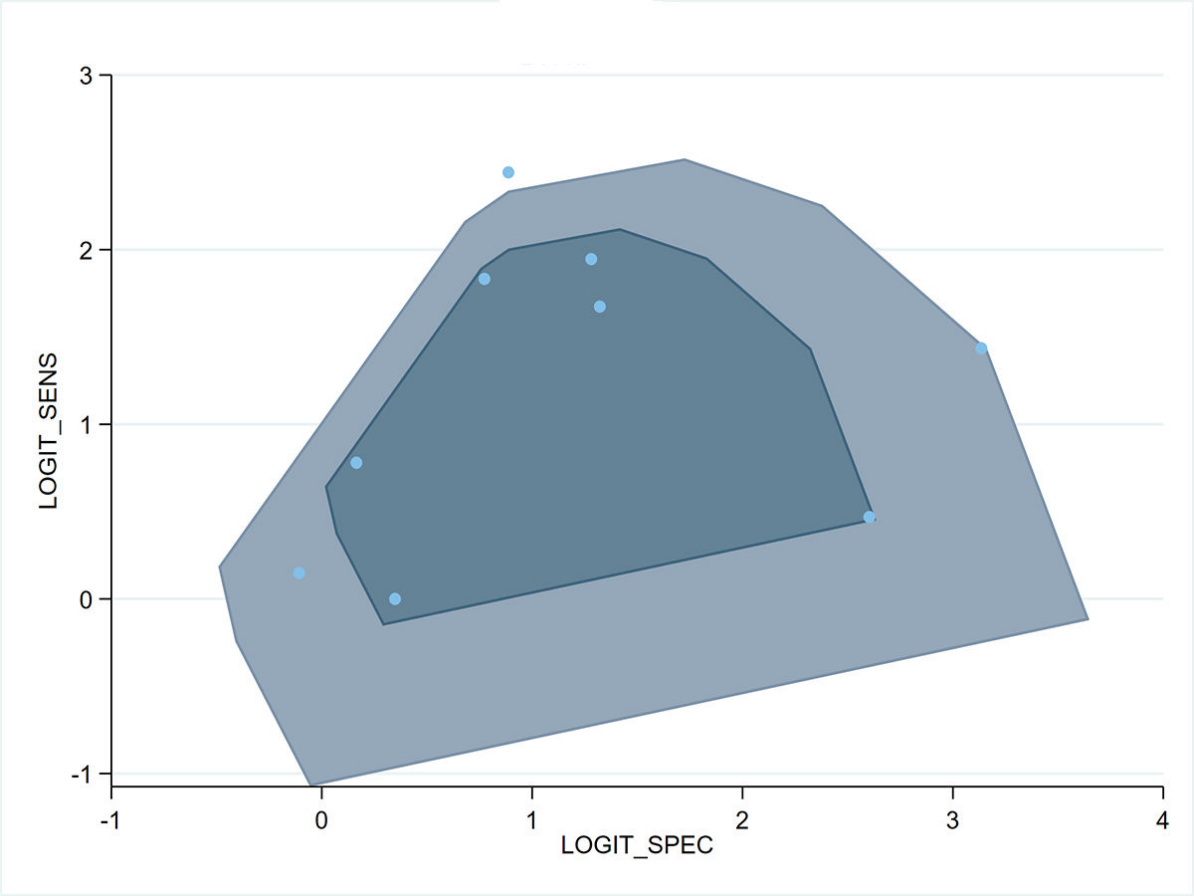
Bivariate boxplot of the combined logit-transformed sensitivity (SENS) and specificity (SPEC) estimates from the bivariate random-effects meta-analysis for predicting treatment response in patients with anxiety disorders after cognitive behavioral therapy. Squares represent individual study estimates; the box illustrates the joint distribution.

The pooled sensitivity of AI models was 0.73 (95% CI 0.58‐0.85; 95% PI 0.31‐0.99; *I*²=82.8%), specificity was 0.75 (95% CI 0.59‐0.89; 95% PI 0.25‐1.00; *I*²=96.7%), accuracy was 0.74 (95% CI 0.62‐0.84; 95% PI 0.39‐0.97; *I*²=94.6%), and AUC was 0.81 (95% CI 0.78‐0.85), as shown in Figures 4-7. Based on a prespecified pretest probability of 20%, the Fagan nomogram indicated a posttest probability of 43% for a positive result and 8% for a negative result (Figure S1 in Multimedia Appendix 1). The results of the bivariate boxplots indicated that 1 study [30] had discrete values, suggesting it may be a source of heterogeneity (Figure 8). Furthermore, meta-regression analysis indicated that heterogeneity in sensitivity was significantly associated with the number of centers (*P*<.001) and the type of anxiety disorder (*P*<.001). In contrast, heterogeneity in specificity was significantly associated with CBT type (*P*<.001) and AI algorithm category (*P*<.001) (Table 2).

**Table 2.** Results of the univariable meta-regression analysis examining potential sources of heterogeneity in the performance of artificial intelligence models for predicting cognitive behavioral therapy treatment response in anxiety disorders.

Subgroup	Number of contingency tables	Sensitivity, value (95% CI)	Meta-regression *P* value	Specificity, value (95% CI)	Meta-regression *P* value
Number of centers			.02		.89
Single center	2	0.87 (0.76‐0.99)		0.74 (0.46‐1.00)	
Multiple centers	7	0.69 (0.58‐0.80)		0.76 (0.61‐0.90)	
Type of anxiety disorder			.01		.10
SAD^a^	3	0.86 (0.74‐0.98)		0.87 (0.71‐1.00)	
Others	6	0.68 (0.57‐0.79)		0.69 (0.54‐0.85)	
Data splitting method			.23		.28
LOOCV^b^	4	0.78 (0.62‐0.93)		0.81 (0.63‐0.98)	
Independent validation	5	0.72 (0.58‐0.86)		0.71 (0.54‐0.89)	
CBT^c^ type			.77		<.001
iCBT^d^	2	0.71 (0.48‐0.94)		0.95 (0.91‐0.98)	
Standard CBT	6	0.74 (0.62‐0.87)		0.61 (0.51‐0.71)	
AI^e^ algorithms			.51		<.001
DL^f^	1	0.62 (0.29‐0.94)		0.93 (0.87‐1.00)	
ML^g^	8	0.78 (0.68‐0.88)		0.67 (0.57‐0.78)	

a SAD: social anxiety disorder.

b LOOCV: leave-one-out cross-validation.

c CBT: cognitive behavioral therapy.

d iCBT: internet-based cognitive behavioral therapy.

e AI: artificial intelligence‌.

f DL: deep learning.

g ML: machine learning.

### Subgroup Analysis

Within the data-type subgroup, multimodal AI models appeared to achieve higher pooled sensitivity (0.84, 95% CI 0.60‐0.97) and accuracy (0.82, 95% CI 0.66‐0.92) compared with single-modal approaches. In contrast, models based on either clinical/demographic or imaging data alone demonstrated comparatively lower sensitivity and accuracy. Regarding specificity, models developed using clinical/demographic data showed relatively higher estimates (0.81, 95% CI 0.55‐0.93). Regarding AUC, only 1 study reported data within this subgroup, and in that study, imaging models achieved an AUC of 0.79 (95% CI 0.76‐0.83) (Table 3).

**Table 3.** Subgroup meta-analysis of the pooled predictive performance of artificial intelligence models for predicting cognitive behavioral therapy response, stratified by input data type, algorithm type, and anxiety disorder classification.

Subgroup	Number of contingency tables	Sensitivity, value (95% CI)	Specificity, value (95% CI)	AUC^a^, value (95% CI)	Accuracy, value (95% CI)
Type of data					
Clinical/demographic	2	0.65 (0.42‐0.82)	0.81 (0.55‐0.93)	—^b^	0.73 (0.55‐0.89)
Imaging	6	0.77 (0.63‐0.87)	0.71 (0.53‐0.85)	0.79 (0.76‐0.83)	0.72 (0.00‐1.00)
Multimodal	1	0.84 (0.60‐0.97)	0.79 (0.54‐0.94)	—	0.82 (0.66‐0.92)
AI^c^ algorithms					
RF^d^	3	0.64 (0.58‐0.70)	0.52 (0.45‐0.59)	0.54 (0.50‐0.59)	0.63 (0.27‐0.93)
SVM^e^	4	0.82 (0.61‐0.93)	0.75 (0.60‐0.85)	0.83 (0.80‐0.86)	0.78 (0.50‐0.97)
RNN^f^	1	0.62 (0.60‐0.63)	0.93 (0.92‐0.94)	—	0.81 (0.81‐0.82)
LR^g^	1	0.84 (0.60‐0.97)	0.79 (0.54‐0.94)	—	0.82 (0.66‐0.92)
Type of anxiety disorder					
GAD^h^ and PD^i^	1	0.86 (0.68‐0.96)	0.68 (0.43‐0.87)	—	0.79 (0.65‐0.90)
SAD^j^	3	0.86 (0.78‐0.92)	0.87 (0.41‐1.00)	0.92 (0.85‐0.98)	0.85 (0.72‐0.94)
PD with AG^k^	2	0.69 (0.00‐1.00)	0.71 (0.00‐1.00)	—	0.69 (0.00‐1.00)
Multiple anxiety disorders	3	0.61 (0.45‐0.77)	0.68 (0.05‐1.00)	0.65 (0.49‐0.81)	0.65 (0.25‐0.96)

a AUC: area under curve.

b Not applicable.

c AI: artificial intelligence.

d RF: random forest.

e SVM: support vector machine.

f RNN: recurrent neural network.

g LR: logistic regression.

h GAD: generalized anxiety disorder.

i PD: panic disorder.

j SAD: social anxiety disorder.

k AG: agoraphobia.

Within the AI algorithm subgroup, different algorithms demonstrated distinct advantages across various performance metrics. For sensitivity, LR performed best, achieving a value of 0.84 (95% CI 0.60‐0.97). Similarly, in terms of accuracy, LR again showed optimal performance, reaching a value of 0.82 (95% CI 0.66‐0.92). For specificity, the RNN achieved the highest value of 0.93 (95% CI 0.87‐0.98). Regarding the AUC, only 2 algorithms produced results that could be combined, with the SVM algorithm yielding a relatively high combined AUC of 0.83 (95% CI 0.80‐0.86).

Within the type of anxiety disorder subgroup, predictive performance varied across outcome metrics. For sensitivity, GAD and PD demonstrated relatively higher pooled estimates at 0.86 (95% CI 0.68‐0.96). In contrast, SAD showed comparatively stronger performance in specificity and accuracy, with a pooled specificity of 0.87 (95% CI 0.41‐1.00) and accuracy of 0.85 (95% CI 0.72‐0.94). For discrimination, as measured by AUC, pooled estimates were available for 2 disorder types, among which SAD demonstrated a comparatively higher AUC of 0.92 (95% CI 0.85‐0.98).

### Publication Bias

Funnel plots and Egger regression tests were conducted to evaluate potential small-study effects (Figures S2-S4 in Multimedia Appendix 1). No statistically significant evidence of small-study effects was observed for sensitivity (*t_7_*=1.75; *P*=.12; intercept=1.54; SE=0.88) and accuracy (*t*_7_=−1.51; *P*=.17; intercept=−2.40; SE=1.59). In contrast, the test for specificity yielded a statistically significant result (*t*_7_=−2.58; *P*=.04; intercept=−4.29; SE=1.66), suggesting the possible presence of small-study effects.

## Discussion

### Principal Findings

The systematic review and meta-analysis revealed that the model demonstrated moderate predictive performance in forecasting treatment response to CBT among patients with anxiety disorders, with sensitivity, specificity, accuracy, and AUC values of 0.73, 0.75, 0.74, and 0.81, respectively. This moderate predictive capability reflects the combined influence of multiple complex factors. First, limitations in input variables constitute a key constraint: current models primarily rely on demographic data, psychiatric history, and scale measurements, making it challenging to capture deeper biological and environmental factors [7,35]. While biomarkers, such as neuroimaging, could theoretically significantly enhance predictive accuracy, their clinical application remains constrained by cost and accessibility [32,35]. Second, data heterogeneity and sample size issues cannot be overlooked. Significant variations among patient cohorts in symptom severity, comorbid conditions, and treatment modalities diminish the model’s generalizability [18,36]. Small-sample studies often overestimate predictive capability, while large-scale multicenter studies frequently yield reduced accuracy, sometimes approaching random levels [18,35].

Furthermore, limitations exist in variable selection and modeling approaches: prediction capabilities relying solely on traditional questionnaires and demographic data are constrained [35,37]. While integrating multimodal data (eg, neuroimaging, genetic, and behavioral data) may enhance performance, the comprehensive collection of such data remains challenging in real-world clinical settings [18]. Finally, challenges in practical application and external validation cannot be overlooked; many models lack independent external validation, resulting in significantly reduced accuracy and interpretability in clinical settings [38]. In summary, while current models demonstrate predictive potential, future research requires larger-scale, multicenter, and multimodal studies to enhance model robustness and clinical translation capabilities.

The subgroup analysis suggested that multimodal data integration was associated with comparatively higher sensitivity (0.84) and accuracy (0.82) than single-modal approaches. Across algorithm categories, no single model consistently outperformed others; LR showed higher sensitivity (0.84) and accuracy (0.82), RNN demonstrated higher specificity (0.93), and SVM had a comparatively higher AUC of 0.83. Predictive performance also varied across anxiety disorder subtypes. GAD and PD showed comparatively higher sensitivity (0.86), whereas SAD demonstrated higher specificity (0.87), accuracy (0.85), and AUC (0.92). Together, these findings suggest that optimal prediction may depend less on a single “best” algorithm and more on strategically matching the model architecture to the data type and clinical question [39]. Future work should therefore focus not only on multimodal data collection but also on developing tailored, interpretable modeling approaches that can be validated in real-world clinical settings [40].

The systematic review revealed that multimodal data demonstrated the most promising performance in predicting CBT treatment response for anxiety disorders, with a sensitivity of 0.84 and an accuracy of 0.82. The superior predictive capability of multimodal approaches could be attributed to several key mechanisms. First, multimodal data integration enabled the comprehensive capture of the multidimensional nature of anxiety disorders by combining neuroimaging, physiological signals, behavioral data, and clinical assessments [29,30]. Complementary information across modalities enabled AI models to detect nuanced individual differences and potential mechanisms of treatment response [27,34]. Research indicates that, compared with single-modality approaches, multimodal fusion significantly enhances predictive accuracy, particularly when incorporating models that integrate functional and structural brain connectivity [29,34]. Moreover, multimodal approaches supported personalized, dynamic prediction models that can identify high-risk nonresponders early, potentially facilitating adaptive treatment strategies [17,27]. While these findings are promising, careful validation through larger, cross-validated studies is essential to establish the clinical utility and generalizability of such AI-driven prediction models [29,33].

The meta-analysis revealed differential performance patterns across ML algorithms in predicting CBT treatment response for anxiety disorders. LR demonstrated comparatively higher sensitivity (0.84) and accuracy (0.82), potentially attributable to its ability to capture linear relationships between clinical features and treatment outcomes [7,31]. The algorithm’s strength lies in its ability to model structured clinical data with minimal risk of overfitting, particularly in scenarios with balanced sample distributions [7,17]. Conversely, RNNs exhibited superior specificity (0.93), likely due to their ability to process high-dimensional, sequential data and capture complex nonlinear interactions [17]. RNN’s advanced modeling approach enables more nuanced identification of nonresponders by leveraging temporal patterns in clinical measurements and platform interaction behaviors [17]. For overall discrimination, SVM achieved a pooled AUC of 0.83 (95% CI 0.80‐0.86). Given their kernel-based formulation, SVMs may perform well with moderate sample sizes and complex feature spaces, allowing flexible decision boundaries [7,31]. Taken together, these findings suggest that algorithmic performance may vary according to data structure and modeling objectives. Rather than indicating a universally superior approach, the results support the importance of aligning model architecture with data characteristics and specific clinical prediction goals [31].

The subgroup analysis indicated that predictive performance varied across anxiety disorder subtypes. GAD and PD demonstrated comparatively higher sensitivity estimates (0.86, 95% CI 0.68‐0.96), suggesting relatively stronger identification of treatment responders in these populations. In contrast, SAD showed higher pooled specificity (0.87, 95% CI 0.41‐1.00) and accuracy (0.85, 95% CI 0.72‐0.94). For discrimination as measured by AUC, pooled estimates were available for 2 disorder types, among which SAD demonstrated a comparatively higher AUC of 0.92 (95% CI 0.85‐0.98). The variation in predictive performance across anxiety disorder subtypes may partly account for the observed heterogeneity. In the subgroup analysis, GAD and PD demonstrated higher sensitivity, which may be related to the use of structured symptom-based scales that provide relatively stable responder signals for model training [27]. First, SAD research often relies on symptom measures that closely align core fear and avoidance dimensions with treatment outcome definitions, which may facilitate clearer model discrimination [7,34]. Second, some SAD studies incorporate high-information features beyond routine clinical variables, potentially enhancing overall predictive performance [29,32]. Third, SAD samples are frequently drawn from relatively homogeneous clinical trials with standardized intervention protocols, which may reduce heterogeneity and improve model stability [7,32].

### Comparison With Prior Work

In the systematic review and meta-analysis published by Vieira et al [35] in 2022, the authors evaluated ML approaches for predicting CBT treatment response across multiple psychiatric disorders. Their analysis included 24 studies comprising 7497 patients and covered 5 diagnostic groups: major depressive disorder, obsessive-compulsive disorder (OCD), posttraumatic stress disorder (PTSD), anxiety disorders, and substance use disorders. The overall pooled accuracy was 0.74 (95% CI 0.70‐0.78), with variability across diagnostic categories. PTSD, anxiety disorders, and OCD demonstrated comparatively higher accuracy estimates (0.787, 0.776, and 0.761, respectively). In comparison, our study focused specifically on anxiety disorders and yielded a pooled accuracy of 0.74 (95% CI 0.62‐0.84), which is comparable to the overall estimate reported by Vieira et al [35]. However, our analysis extended beyond accuracy by systematically synthesizing sensitivity, specificity, and AUC to provide a more comprehensive assessment of predictive performance. In addition, we conducted subgroup analyses examining the potential influence of data types (eg, clinical and neuroimaging), algorithm types, and disease types on model performance, thereby offering a more granular evaluation within the anxiety disorder domain. Bubble plots were used to illustrate temporal trends and the distribution of study characteristics across performance metrics.

### Heterogeneity

The substantial heterogeneity observed across studies may influence the overall strength of evidence for AI-based prediction models. Given the anticipated between-study variability, a bivariate random-effects model was applied to pool sensitivity and specificity estimates. Meta-regression and subgroup analyses were further conducted to explore potential sources of heterogeneity. The results indicated that heterogeneity in sensitivity was significantly associated with the number of study centers (*P*=.02) and the anxiety disorder subtype (*P*=.01). In contrast, heterogeneity in specificity was significantly associated with CBT type (*P*<.001) and AI algorithm category (*P*<.001). The comparatively higher sensitivity observed in single-center studies, compared with multicenter studies, may reflect more homogeneous samples and more consistent data collection procedures [41]. Differences across anxiety disorder types may relate to variations in symptom structure, measurement alignment, and sample characteristics, which could influence the clarity of the responder signals available to the models [42]. Meanwhile, differences between iCBT and standard CBT may reflect variations in intervention structure and in the intensity of monitoring [43]. Variability in specificity across algorithm categories (DL vs ML) may relate to differences in model complexity and data representation strategies [44].

The bivariate boxplot suggested that 1 study [30] may represent a potential source of heterogeneity. Nevertheless, heterogeneity is likely multifactorial. Additional contributors may include differences in patient age, disorder severity, prior treatment exposure, geographic setting, sample size, feature selection strategies, data preprocessing methods, and hyperparameter optimization [45-48]. The combined influence of these methodological and clinical factors may account for the variability in predictive performance. Future research would benefit from more standardized reporting and systematic consideration of these variables to enhance reproducibility and external validity.

### Implications for Practice and Research

Discussing the clinical interpretation of our results, the AI models demonstrated moderate predictive value for CBT treatment response in anxiety disorders, with the strongest performance observed for SAD. Multimodal models, which combine clinical demographics with imaging data, showed relatively superior predictive performance compared with unimodal approaches. This suggests that AI could support clinicians in making earlier, more informed therapeutic decisions and potentially improve patient outcomes by enabling timely treatment adjustments [49,50]. Specifically, these predictive models could be integrated into clinical workflows at key decision points: (1) during initial assessment to help stratify patients by likelihood of response, informing the intensity of early monitoring or the consideration of augmented treatment protocols, and (2) during the early stages of CBT to identify potential nonresponders, allowing for a timely switch or integration of other evidence-based interventions before disengagement occurs [35,51].

However, AI should serve as a decision-support tool rather than replacing clinician judgment, since treatment choices for anxiety disorders are influenced by not only severity but also patient preferences, comorbidity, and functional goals [52]. In the treatment of anxiety disorders, complex therapeutic decisions not only depend on assessing disease severity but also closely relate to the patient’s individual condition and needs [53]. For real-world deployment, developing AI tools as clinician-facing dashboards that present predictive probabilities alongside key clinical context, rather than as autonomous decision systems, is crucial for fostering trust and facilitating shared decision-making [54]. Notably, among the studies we included, external validation sets were relatively scarce, and no research has yet focused solely on predicting outcomes for external validation cohorts. Future studies are needed to evaluate the generalization capabilities of AI models. Furthermore, limitations to the future adoption of AI include scarce annotated anxiety disorder data and regulatory hurdles [55]. Technical challenges persist in data availability, model interpretability, and transparency [56]. Advances in few-shot learning, self-supervised models, and centralized platforms may support an integrated AI ecosystem [57].

### Limitations

When interpreting the findings of this systematic review and meta-analysis on the AI prediction of CBT treatment response for anxiety disorders, several limitations should be considered. First, the PROBAST+AI assessment indicated a high risk of bias in over half of the included studies, particularly in analytical methodology, which may have influenced the pooled estimates and contributed to between-study heterogeneity. Future studies should adopt more rigorous design and transparent reporting standards. Second, variability in the definition of treatment response across studies may have affected comparability. Although most scales assessed anxiety symptom reduction, differences in operational criteria could influence predictive performance. Greater standardization of outcome definitions would enhance consistency in future research. Third, the substantial heterogeneity and wide PIs suggest that model performance may vary considerably across clinical settings. Therefore, the pooled estimates should be interpreted cautiously and regarded as preliminary within the context of current methodological constraints. Fourth, although heterogeneity was observed across studies, this likely reflects differences in study design, patient characteristics, and modeling approaches. The reported PIs indicate that performance may vary across settings, highlighting the importance of external validation in future research. Finally, due to limited data availability, direct head-to-head comparisons between AI models and routinely used clinical scales were not feasible. Future research should incorporate comparative designs to better assess the incremental value of AI-based prediction tools. Overall, while AI models demonstrate potential for supporting treatment decision-making, stronger evidence from externally validated and methodologically robust studies is needed before clinical implementation.

### Conclusions

This meta-analysis quantitatively synthesized AI performance in predicting CBT response for anxiety disorders, moving beyond narrative reviews to provide pooled evidence. In contrast to existing reviews that encompass broader diagnostic groups, our focused approach establishes a precise benchmark for this clinical domain, highlighting the current moderate overall performance. Furthermore, we extend beyond previous work by demonstrating the superior predictive utility of multimodal data, identifying SAD as the most predictable subtype, and systematically evaluating the impact of data modalities and algorithm types. Future efforts should prioritize robustly validated multimodal models, laying essential groundwork for the potential development of AI-assisted tools to personalize treatment planning in anxiety disorders.

## Supplementary material

10.2196/86079 Multimedia Appendix 1 Data to support the study.

10.2196/86079 Multimedia Appendix 2 Technical aspects and diagnostic performance data extracted from the included studies.

10.2196/86079 Checklist 1 PRISMA-DTA checklist.

10.2196/86079 Checklist 2 PRISMA 2020 Abstract checklist

10.2196/86079 Checklist 3 PRISMA-S checklist

## Abbreviations

AI: artificial intelligence
AUC: area under the curve
CBT: cognitive behavioral therapy
CGI-I: Clinical Global Impressions–Improvement
DL: deep learning
FN: false negative
FP: false positive
GAD: generalized anxiety disorder
GAD-7: Generalized Anxiety Disorder-7
HAM-A: Hamilton Anxiety Rating Scale
HKSJ: Hartung-Knapp-Sidik-Jonkman
LR: logistic regression
LSAS: Liebowitz Social Anxiety Scale
ML: machine learning
OASIS: Overall Anxiety Severity and Impairment Scale
OCD: obsessive-compulsive disorder
PD: panic disorder
PHQ-9: Patient Health Questionnaire-9
PI: prediction interval
PRISMA: Preferred Reporting Items for Systematic Reviews and Meta-Analyses
PROBAST+AI: Prediction Model Risk of Bias Assessment Tool for Artificial Intelligence
PTSD: posttraumatic stress disorder
RF: random forest
RNN: recurrent neural network
SAD: social anxiety disorder
SPQ: Spider Phobia Questionnaire
SVM: support vector machine
TN: true negative
TP: true positive
