# Supplementary material for: Artificial Intelligence for Predicting Treatment Response in Patients With Anxiety Disorders After Cognitive Behavioral Therapy: Systematic Review and Meta-Analysis
Source: J Med Internet Res. 2026 Mar 18;28:e86079. doi: 10.2196/86079 (PMC12998711; doi:10.2196/86079)

Table S1. Search strategy in PubMed, Embase, Web of Science, Cochrane Library, and PsycINFO.

| Database | Search strategy | Filters and Limits | Number of Studies |
| --- | --- | --- | --- |
| PubMed | ("Cognitive Behavioral Therapy"[Mesh] OR "Cognitive Behavioral Therapy"[tiab] OR "Cognitive Behavior Therapy"[tiab] OR "Cognitive Behaviour Therapy"[tiab] OR "Cognitive Psychotherapy"[tiab] OR "Cognitive Therapies"[tiab] OR "Cognitive Behavior Therapies"[tiab] OR "Cognitive Behaviour Therapies"[tiab] OR "Behaviour Therapy, Cognitive"[tiab] OR “CBT”[tiab])  AND  ("Artificial Intelligence"[Mesh] OR "Machine Learning"[Mesh] OR "Deep Learning"[Mesh] OR "Support Vector Machine"[Mesh] OR "Machine Learning"[tiab] OR "Deep Learning"[tiab] OR "Artificial Intelligence"[tiab] OR "AI"[tiab] OR "Computer Reasoning"[tiab] OR "Machine Intelligence"[tiab] OR "Prediction model"[tiab] OR “Predictive model”[tiab] OR "SVM"[tiab] OR "Support Vector Machine"[tiab] OR "Logistic Regression"[tiab] OR "Decision Tree"[tiab] OR "Random Forest"[tiab] OR "Neural Network"[tiab] OR "K-Nearest Neighbors"[tiab])  AND  ("Anxiety Disorders"[Mesh] OR "Phobia, Social"[Mesh] OR "Generalized Anxiety Disorder"[Mesh] OR "Panic Disorder"[Mesh] OR "Anxiety, Separation"[Mesh] OR "anxiety disorders"[tiab] OR "generalized anxiety disorder"[tiab] OR "social anxiety disorder"[tiab] OR "social phobia"[tiab] OR "panic disorder"[tiab] OR "specific phobia"[tiab] OR "agoraphobia"[tiab] OR "Separation Anxiety"[tiab] OR "separation anxiety disorder"[tiab] OR "selective mutism"[tiab] OR "substance induced anxiety disorder"[tiab] OR "phobic disorders"[tiab] OR "panic attacks"[tiab] OR "anxiety symptoms"[tiab] OR "anxiety treatment"[tiab] OR "Anxiety Disorder, Generalized"[tiab] OR "Anxiety Disorders, Generalized"[tiab] OR "Disorder, Generalized Anxiety"[tiab] OR "Disorders, Generalized Anxiety"[tiab] OR "Generalized Anxiety Disorders"[tiab] OR "Generalized Anxiety"[tiab] OR "Anxiety, Generalized"[tiab] OR "Generalized Anxieties"[tiab] OR "General Anxiety Disorder"[tiab] OR "Anxiety Disorder, General"[tiab] OR "Anxiety Disorders, General"[tiab] OR "Disorder, General Anxiety"[tiab] OR "Disorders, General Anxiety"[tiab] OR "General Anxiety Disorders"[tiab] OR "Overanxious Disorder"[tiab] OR "Disorder, Overanxious"[tiab] OR "Disorders, Overanxious"[tiab] OR "Overanxious Disorders"[tiab] OR "Anxiety Disorder"[tiab] OR "Disorder, Anxiety"[tiab] OR "Disorders, Anxiety"[tiab] OR "Neuroses, Anxiety"[tiab] OR "Anxiety Neuroses"[tiab] OR "Anxiety States, Neurotic"[tiab] OR "Neurotic Anxiety States"[tiab] OR "Social Phobia"[tiab] OR "Social Phobias"[tiab] OR "Social Anxiety Disorder"[tiab] OR "Anxiety Disorder, Social"[tiab] OR "Disorder, Social Anxiety"[tiab] OR "Social Anxiety Disorders"[tiab] OR "Sociophobia"[tiab] OR "Fear of Social Evaluation"[tiab]) | No restrictions applied regarding date range, language, or publication type. | 168 |
| Embase | ('cognitive behavioral therapy'/exp OR 'Cognitive Behavioral Therapy':ab,ti OR 'Cognitive Behavior Therapy':ab,ti OR 'Cognitive Behaviour Therapy':ab,ti OR 'Cognitive Psychotherapy':ab,ti OR 'Cognitive Therapies':ab,ti OR 'Cognitive Behavior Therapies':ab,ti OR 'Cognitive Behaviour Therapies':ab,ti OR 'Behaviour Therapy, Cognitive':ab,ti OR 'CBT':ab,ti)  AND  ('Artificial Intelligence'/exp OR 'Machine Learning'/exp OR 'Deep Learning'/exp OR 'Support Vector Machine'/exp OR 'Machine Learning':ab,ti OR 'Deep Learning':ab,ti OR 'Artificial Intelligence':ab,ti OR 'AI':ab,ti OR 'Computer Reasoning':ab,ti OR 'Machine Intelligence':ab,ti OR 'Prediction model':ab,ti OR 'Predictive model':ab,ti OR 'SVM':ab,ti OR 'Support Vector Machine':ab,ti OR 'Logistic Regression':ab,ti OR 'Decision Tree':ab,ti OR 'Random Forest':ab,ti OR 'Neural Network':ab,ti OR 'K-Nearest Neighbors':ab,ti)  AND  ('anxiety disorder'/exp OR 'social phobia'/exp OR 'generalized anxiety disorder'/exp OR 'separation anxiety'/exp OR 'panic'/exp OR 'anxiety disorders':ab,ti OR 'generalized anxiety disorder':ab,ti OR 'social anxiety disorder':ab,ti OR 'social phobia':ab,ti OR 'panic disorder':ab,ti OR 'specific phobia':ab,ti OR 'agoraphobia':ab,ti OR 'Separation Anxiety':ab,ti OR 'separation anxiety disorder':ab,ti OR 'selective mutism':ab,ti OR 'substance induced anxiety disorder':ab,ti OR 'phobic disorders':ab,ti OR 'panic attacks':ab,ti OR 'anxiety symptoms':ab,ti OR 'anxiety treatment':ab,ti OR 'Anxiety Disorder, Generalized':ab,ti OR 'Anxiety Disorders, Generalized':ab,ti OR 'Disorder, Generalized Anxiety':ab,ti OR 'Disorders, Generalized Anxiety':ab,ti OR 'Generalized Anxiety Disorders':ab,ti OR 'Generalized Anxiety':ab,ti OR 'Anxiety, Generalized':ab,ti OR 'Generalized Anxieties':ab,ti OR 'General Anxiety Disorder':ab,ti OR 'Anxiety Disorder, General':ab,ti OR 'Anxiety Disorders, General':ab,ti OR 'Disorder, General Anxiety':ab,ti OR 'Disorders, General Anxiety':ab,ti OR 'General Anxiety Disorders':ab,ti OR 'Overanxious Disorder':ab,ti OR 'Disorder, Overanxious':ab,ti OR 'Disorders, Overanxious':ab,ti OR 'Overanxious Disorders':ab,ti OR 'Anxiety Disorder':ab,ti OR 'Disorder, Anxiety':ab,ti OR 'Disorders, Anxiety':ab,ti OR 'Neuroses, Anxiety':ab,ti OR 'Anxiety Neuroses':ab,ti OR 'Anxiety States, Neurotic':ab,ti OR 'Neurotic Anxiety States':ab,ti OR 'Social Phobia':ab,ti OR 'Social Phobias':ab,ti OR 'Social Anxiety Disorder':ab,ti OR 'Anxiety Disorder, Social':ab,ti OR 'Disorder, Social Anxiety':ab,ti OR 'Social Anxiety Disorders':ab,ti OR 'Sociophobia':ab,ti OR 'Fear of Social Evaluation':ab,ti) | No restrictions applied regarding date range, language, or publication type. | 599 |
| Web of Science | TS=("Cognitive Behavioral Therapy" OR "Cognitive Behavior Therapy" OR "Cognitive Behaviour Therapy" OR "Cognitive Psychotherapy" OR "Cognitive Therapies" OR "Cognitive Behavior Therapies" OR "Cognitive Behaviour Therapies" OR "Behaviour Therapy Cognitive" OR "CBT") AND  TS=("Artificial Intelligence" OR "Machine Learning" OR "Deep Learning" OR "Support Vector Machine" OR "AI" OR "Computer Reasoning" OR "Machine Intelligence" OR "Prediction model" OR "Predictive model" OR "SVM" OR "Logistic Regression" OR "Decision Tree" OR "Random Forest" OR "Neural Network" OR "K-Nearest Neighbors")  AND  TS=("anxiety disorders" OR "social anxiety disorder" OR "social phobia" OR "panic disorder" OR "specific phobia" OR "agoraphobia" OR "separation anxiety" OR "separation anxiety disorder" OR "selective mutism" OR "substance induced anxiety disorder" OR "phobic disorders" OR "panic attacks" OR "anxiety symptoms" OR "anxiety treatment" OR "generalized anxiety disorder" OR "generalized anxiety" OR "anxiety generalized" OR "generalized anxieties" OR "general anxiety disorder" OR "anxiety disorder general" OR "general anxiety disorders" OR "overanxious disorder" OR "disorder overanxious" OR "overanxious disorders" OR "anxiety disorder" OR "disorder anxiety" OR "neuroses anxiety" OR "anxiety neuroses" OR "anxiety states neurotic" OR "neurotic anxiety states" OR "social phobias" OR "anxiety disorder social" OR "disorder social anxiety" OR "social anxiety disorders" OR "sociophobia" OR "fear of social evaluation") | No restrictions applied regarding date range, language, or publication type. | 326 |
| Cochrane Library | ("Cognitive Behavioral Therapy" OR "Cognitive Behavior Therapy" OR "Cognitive Behaviour Therapy" OR "Cognitive Psychotherapy" OR "Cognitive Therapies" OR "Cognitive Behavior Therapies" OR "Cognitive Behaviour Therapies" OR "Behaviour Therapy Cognitive" OR "CBT") AND  ("Artificial Intelligence" OR "Machine Learning" OR "Deep Learning" OR "Support Vector Machine" OR "AI" OR "Computer Reasoning" OR "Machine Intelligence" OR "Prediction model" OR "Predictive model" OR "SVM" OR "Logistic Regression" OR "Decision Tree" OR "Random Forest" OR "Neural Network" OR "K-Nearest Neighbors")  AND  ("anxiety disorders" OR "social anxiety disorder" OR "social phobia" OR "panic disorder" OR "specific phobia" OR "agoraphobia" OR "separation anxiety" OR "separation anxiety disorder" OR "selective mutism" OR "substance induced anxiety disorder" OR "phobic disorders" OR "panic attacks" OR "anxiety symptoms" OR "anxiety treatment" OR "generalized anxiety disorder" OR "generalized anxiety" OR "anxiety generalized" OR "generalized anxieties" OR "general anxiety disorder" OR "anxiety disorder general" OR "general anxiety disorders" OR "overanxious disorder" OR "disorder overanxious" OR "overanxious disorders" OR "anxiety disorder" OR "disorder anxiety" OR "neuroses anxiety" OR "anxiety neuroses" OR "anxiety states neurotic" OR "neurotic anxiety states" OR "social phobias" OR "anxiety disorder social" OR "disorder social anxiety" OR "social anxiety disorders" OR "sociophobia" OR "fear of social evaluation") | No restrictions applied regarding date range, language, or publication type. | 134 |
| PsycINFO | ("Cognitive Behavioral Therapy" OR "Cognitive Behavior Therapy" OR "Cognitive Behaviour Therapy" OR "Cognitive Psychotherapy" OR "Cognitive Therapies" OR "Cognitive Behavior Therapies" OR "Cognitive Behaviour Therapies" OR "Behaviour Therapy Cognitive" OR "CBT") AND ("Artificial Intelligence" OR "Machine Learning" OR "Deep Learning" OR "Support Vector Machine" OR "AI" OR "Computer Reasoning" OR "Machine Intelligence" OR "Prediction model" OR "Predictive model" OR "SVM" OR "Logistic Regression" OR "Decision Tree" OR "Random Forest" OR "Neural Network" OR "K-Nearest Neighbors") AND ("anxiety disorders" OR "social anxiety disorder" OR "social phobia" OR "panic disorder" OR "specific phobia" OR "agoraphobia" OR "separation anxiety" OR "separation anxiety disorder" OR "selective mutism" OR "substance induced anxiety disorder" OR "phobic disorders" OR "panic attacks" OR "anxiety symptoms" OR "anxiety treatment" OR "generalized anxiety disorder" OR "generalized anxiety" OR "anxiety generalized" OR "generalized anxieties" OR "general anxiety disorder" OR "anxiety disorder general" OR "general anxiety disorders" OR "overanxious disorder" OR "disorder overanxious" OR "overanxious disorders" OR "anxiety disorder" OR "disorder anxiety" OR "neuroses anxiety" OR "anxiety neuroses" OR "anxiety states neurotic" OR "neurotic anxiety states" OR "social phobias" OR "anxiety disorder social" OR "disorder social anxiety" OR "social anxiety disorders" OR "sociophobia" OR "fear of social evaluation") | No restrictions applied regarding date range, language, or publication type. | 172 |

Table S2. Risk of bias assessment (PROBAST+AI) model development based on seven domains.

| Author, year | | Quality | | | | Applicability concerns | | | Overall judgement | |
| --- | --- | --- | --- | --- | --- | --- | --- | --- | --- | --- |
|  |  | Participants and data sources ^a^ | Predictors ^b^ | Outcome ^c^ | Analysis ^d^ | Participants and data sources ^e^ | Predictors ^f^ | Outcome ^g^ | Quality ^h^ | Applicability concerns ^i^ |
| Ball et al [1] | 2014 | L | L | L | H | L | L | L | H | L |
| Bertie et al [2] | 2024 | L | L | L | L | L | L | H | L | H |
| Bukhari et al [3] | 2025 | L | L | L | L | L | L | L | L | L |
| Frick et al [4] | 2020 | L | L | L | H | L | L | L | H | L |
| Hahn et al [5] | 2015 | L | L | L | H | L | L | L | H | L |
| Hilbert et al [6] | 2024 | L | L | L | L | L | L | L | L | L |
| Isacsson et al [7] | 2024 | L | L | L | L | L | L | L | L | L |
| Månsson et al [8] | 2015 | L | L | L | H | L | L | L | H | L |
| Prasad et al [9] | 2023 | L | L | L | L | L | L | L | L | L |
| Sundermann et al [10] | 2017 | L | L | L | H | L | L | L | H | L |
| Whitﬁeld-Gabrieli et al [11] | 2015 | L | L | L | H | L | L | L | H | L |

**Abbreviation:** PROBAST+AI, Prediction model Risk of Bias Assessment Tool + AI, L low; H high; U unclear.

**Footnote:** Signaling questions are rated as "yes" (Y), "probably yes" (PY), "probably no" (PN), "no" (N), "no information" (NI), and in some cases "not applicable" (NA). All signaling questions are phrased in such a way that "yes" or "probably yes" indicates a low risk of bias. Any signaling questions rated as "no" or "probably no" indicate a potential high risk of bias in that domain. If there are no "no" or "probably no" ratings, but "no information" (NI) is present, the risk of bias in that domain is classified as unclear.

a. Participants and data sources

1.1 Were appropriate data sources used?

1.2 Was an appropriate study design used?

1.3 Did the in- and exclusions of study participants result in a representative dataset?

b. Predictors

2.1 Were predictors defined and assessed in a similar way for all participants?

2.2 Was any pre-processing of predictors similar for all participants?

2.3 Were predictor assessments made without knowledge of outcome data?

2.4 Were the predictors included in the model available at the time the model was intended to be used?

c. Outcome

3.1 Were outcomes defined and assessed appropriately?

3.2 Were outcomes defined and assessed in a similar way for all participants?

3.3 Were outcome assessments made without use or knowledge of predictor data?

3.4 Was the time interval between predictor assessment and outcome assessment appropriate?

d. Analysis

4.1 Was there evidence that the sample size was reasonable?

4.2 Were continuous and categorical predictors handled appropriately?

4.3 Were participants with missing or censored data handled appropriately in the analysis?

4.4 If methods to address class imbalance were used, was the model or the model predictions recalibrated?

4.5 Were methods used to address potential model overfitting?

e. Participants and data sources

Concern that the (data of the) included participants do not match the review question or the assessor’s intended use of the prediction model.

f. Predictors

Concern that the definition, pre-processing, assessment, or timing of assessment of the predictors in the model do not match the review question or the assessor’s intended use.

g. Outcome

Concern that the outcome, its definition, assessment, or timing of assessment do not match the review question or the assessor’s intended use.

h. Quality

Low risk: If all four domains were rated low concern regarding quality.

High risk: If at least one domain was rated high concern regarding quality .

Unclear: If at least one domain was rated unclear concern regarding quality and no domains were rated high concern.

i. Applicability concerns

Low risk: If all three domains were rated low concern for applicability.

High risk: If at least one domain was rated high concern for applicability.

Unclear: If at least one domain was rated unclear concern for applicability and no domains were rated high concern.

Table S3. Risk of bias assessment (PROBAST+AI) model evaluation based on seven domains.

| Author, year | | Risk of bias | | | | Applicability concerns | | | Overall judgement | |
| --- | --- | --- | --- | --- | --- | --- | --- | --- | --- | --- |
|  |  | Participants and data sources ^a^ | Predictors ^b^ | Outcome ^c^ | Analysis ^d^ | Participants and data sources ^e^ | Predictors ^f^ | Outcome ^g^ | Risk of bias ^h^ | Applicability concerns ^i^ |
| Ball et al [1] | 2014 | L | L | L | H | L | L | L | H | L |
| Bertie et al [2] | 2024 | L | L | L | L | L | L | H | L | H |
| Bukhari et al [3] | 2025 | L | L | L | L | L | L | L | L | L |
| Frick et al [4] | 2020 | L | L | L | H | L | L | L | H | L |
| Hahn et al [5] | 2015 | L | L | L | H | L | L | L | H | L |
| Hilbert et al [6] | 2024 | L | L | L | L | L | L | L | L | L |
| Isacsson et al [7] | 2024 | L | L | L | L | L | L | L | L | L |
| Månsson et al [8] | 2015 | L | L | L | H | L | L | L | H | L |
| Prasad et al [9] | 2023 | L | L | L | L | L | L | L | L | L |
| Sundermann et al [10] | 2017 | L | L | L | H | L | L | L | H | L |
| Whitﬁeld-Gabrieli et al [11] | 2015 | L | L | L | H | L | L | L | H | L |

**Abbreviation:** PROBAST+AI, Prediction model Risk of Bias Assessment Tool + AI, L low; H high; U unclear.

**Footnote:** Signaling questions are rated as "yes" (Y), "probably yes" (PY), "probably no" (PN), "no" (N), "no information" (NI), and in some cases "not applicable" (NA). All signaling questions are phrased in such a way that "yes" or "probably yes" indicates a low risk of bias. Any signaling questions rated as "no" or "probably no" indicate a potential high risk of bias in that domain. If there are no "no" or "probably no" ratings, but "no information" (NI) is present, the risk of bias in that domain is classified as unclear.

a. Participants and data sources

1.1 Were appropriate data sources used?

1.2 Was an appropriate study design used?

1.3 Did the in- and exclusions of study participants result in a representative dataset?

b. Predictors

2.1 Were predictors defined and assessed in a similar way for all participants?

2.2 Was any pre-processing of predictors similar for all participants?

2.3 Were predictor assessments made without knowledge of outcome data?

2.4 Were the predictors included in the model available at the time the model was intended to be used?

c. Outcome

3.1 Were outcomes defined and assessed appropriately?

3.2 Were outcomes defined and assessed in a similar way for all participants?

3.3 Were outcome assessments made without use or knowledge of predictor data?

3.4 Was the time interval between predictor assessment and outcome assessment appropriate?

d. Analysis

4.1 Was model evaluation based on only apparent performance avoided?

4.2 Was there evidence that the sample size was reasonable?

4.3 Were participants with missing or censored data handled appropriately in the analysis?

4.4 If methods to address class imbalance were used, was the evaluation done in a dataset without imbalance correction?

4.5 If data splitting was done to create training and test datasets, was there evidence that data leakage was avoided?

4.6 If resampling methods were used to evaluate model performance, were all model development steps replicated in the resampling process?

4.7 Was the predictive performance of the model evaluated appropriately, e.g., calibration, discrimination, and net benefit?

e. Participants and data sources

Concern that the (data of the) included participants do not match the review question or the assessor’s intended use of the prediction model.

f. Predictors

Concern that the definition, pre-processing, assessment, or timing of assessment of the predictors in the model do not match the review question or the assessor’s intended use.

g. Outcome

Concern that the outcome, its definition, assessment, or timing of assessment do not match the review question or the assessor’s intended use.

h. Risk of bias

Low risk: If all four domains were rated low risk of bias.

High risk: If at least one domain was rated high risk of bias.

Unclear: If at least one domain was rated unclear risk of bias and no domains were rated high risk of bias.

i. Applicability concerns

Low risk: If all three domains were rated low concern for applicability.

High risk: If at least one domain was rated high concern for applicability.

Unclear: If at least one domain was rated unclear concern for applicability and no domains were rated high concern.

**References**

1. Ball TM, Stein MB, Ramsawh HJ, Campbell-Sills L, Paulus MP. Single-subject anxiety treatment outcome prediction using functional neuroimaging. Neuropsychopharmacology. Apr 2014;39(5):1254-1261. [doi: 10.1038/npp.2013.328]

2. Bertie LA, Quiroz JC, Berkovsky S, et al. Predicting remission following CBT for childhood anxiety disorders: a machine learning approach. Psychol Med. Dec 17, 2024;17:1-11. [doi: 10.1017/S0033291724002654]

3. Bukhari Q, Rosenfield D, Hofmann SG, Gabrieli JDE, Ghosh SS. Predicting treatment response to cognitive behavior therapy in social anxiety disorder on the basis of demographics, psychiatric history, and scales: a machine learning approach. PLoS ONE. 2025;20(3):e0313351. [doi: 10.1371/journal.pone.0313351]

4. Frick A, Engman J, Alaie I, et al. Neuroimaging, genetic, clinical, and demographic predictors of treatment response in patients with social anxiety disorder. J Affect Disord. Jan 15, 2020;261:230-237. [doi: 10.1016/j.jad.2019.10.027]

5. Hahn T, Kircher T, Straube B, et al. Predicting treatment response to cognitive behavioral therapy in panic disorder with agoraphobia by integrating local neural information. JAMA Psychiatry. Jan 2015;72(1):68-74. [doi: 10.1001/jamapsychiatry.2014.1741]

6. Hilbert K, Böhnlein J, Meinke C, et al. Lack of evidence for predictive utility from resting state fMRI data for individual exposure-based cognitive behavioral therapy outcomes: a machine learning study in two large multi-site samples in anxiety disorders. Neuroimage. Jul 15, 2024;295(120639):120639. [doi: 10.1016/j.neuroimage.2024.120639]

7. Hentati Isacsson N, Ben Abdesslem F, Forsell E, Boman M, Kaldo V. Methodological choices and clinical usefulness for machine learning predictions of outcome in Internet-based cognitive behavioural therapy. Commun Med (Lond). Oct 10, 2024;4(1):196. [doi: 10.1038/s43856-024-00626-4]

8. Månsson KNT, Frick A, Boraxbekk CJ, et al. Predicting long-term outcome of Internet-delivered cognitive behavior therapy for social anxiety disorder using fMRI and support vector machine learning. Transl Psychiatry. Mar 17, 2015;5(3):e530. [doi: 10.1038/tp.2015.22]

9. Prasad N, Chien I, Regan T, et al. Deep learning for the prediction of clinical outcomes in internet-delivered CBT for depression and anxiety. PLoS ONE. 2023;18(11):e0272685. [doi: 10.1371/journal.pone.0272685]

10. Sundermann B, Bode J, Lueken U, et al. Support vector machine analysis of functional magnetic resonance imaging of interoception does not reliably predict individual outcomes of cognitive behavioral therapy in panic disorder with agoraphobia. Front Psychiatry. 2017;8:99. [doi: 10.3389/fpsyt.2017.00099]

11. Whitfield-Gabrieli S, Ghosh SS, Nieto-Castanon A, et al. Brain connectomics predict response to treatment in social anxiety disorder. Mol Psychiatry. May 2015;21(5):680-685. [doi: 10.1038/mp.2015.109]

Figure S1: Fagan plot for predicting cognitive behavioral therapy (CBT) treatment response in anxiety disorder patients based on artificial intelligence (AI).


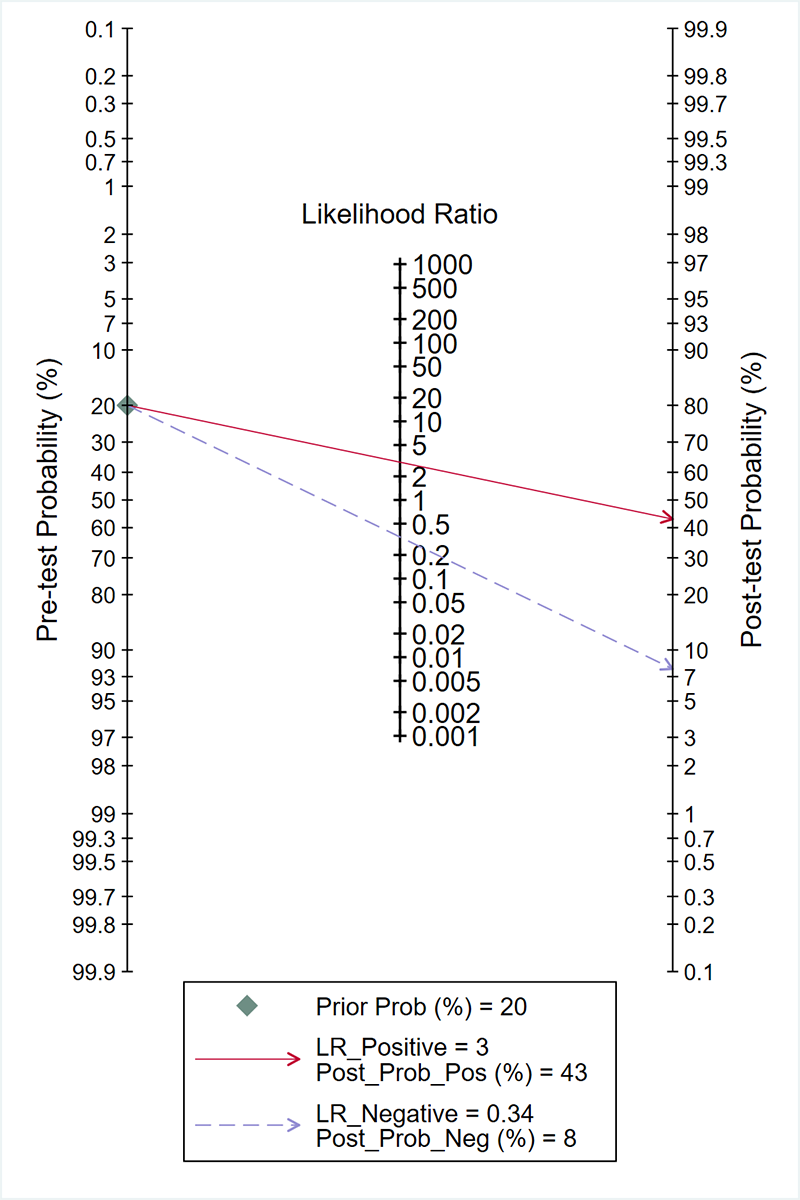


Figure S2: Funnel plot and Egger’s test for publication bias in the sensitivity estimates of AI-based prediction of CBT treatment response in patients with anxiety disorders (*P* < 0.05 considered statistically significant). t = 1.75, df = 7, *P* = 0.12, with an intercept (bias estimate) of 1.54 (SE = 0.88).


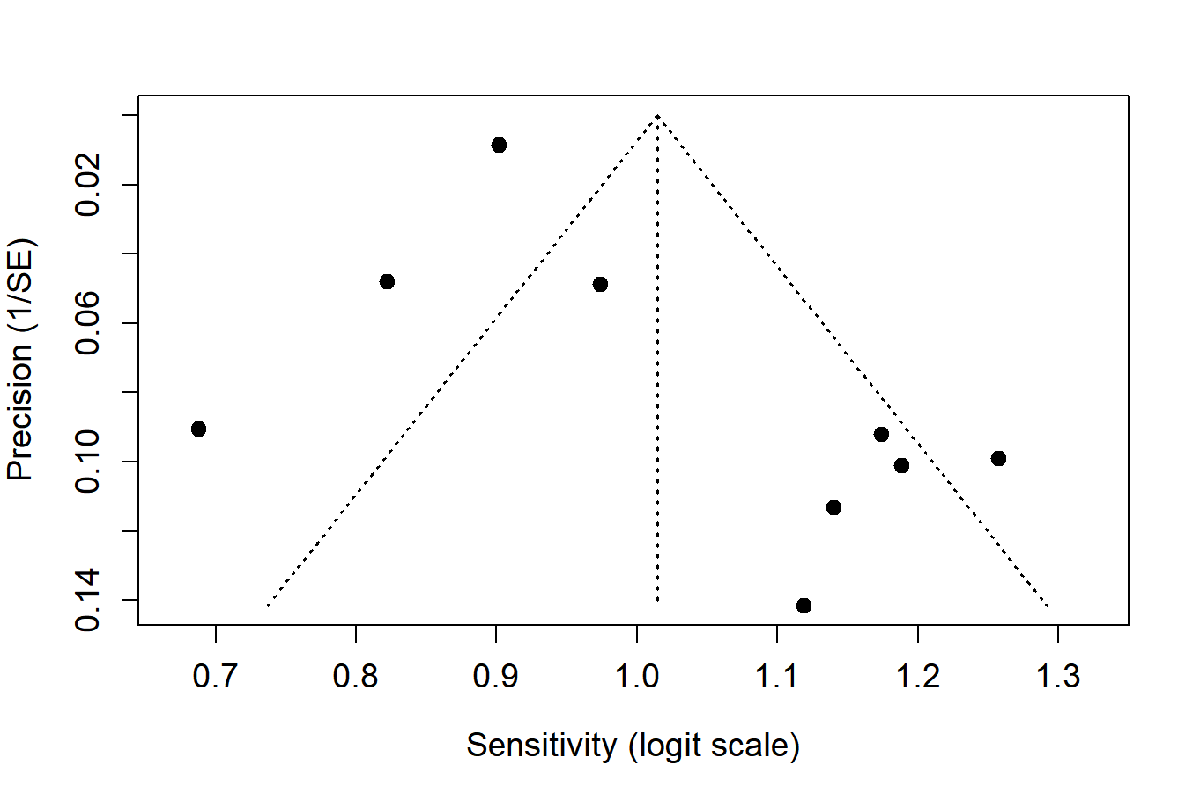


Figure S3: Funnel plot and Egger’s test for publication bias in the specificity estimates of AI-based prediction of CBT treatment response in patients with anxiety disorders (*P* < 0.05 considered statistically significant). t = -2.58, df = 7, *P* = 0.04, with an intercept (bias estimate) of -4.29 (SE = 1.66).


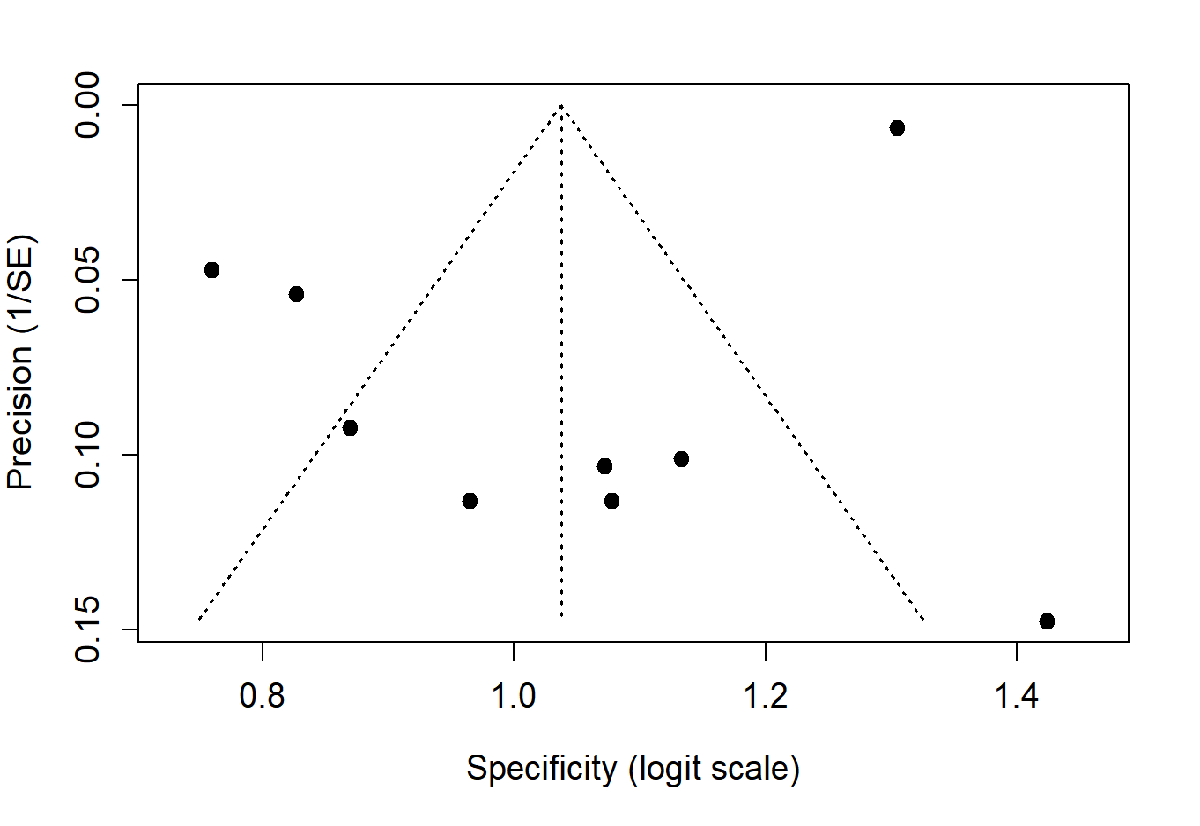


Figure S4: Funnel plot and Egger’s test for publication bias in the accuracy estimates of AI-based prediction of CBT treatment response in patients with anxiety disorders (*P* < 0.05 considered statistically significant). t = -1.51, df = 7, *P* = 0.17, with an intercept (bias estimate) of -2.40 (SE = 1.59).


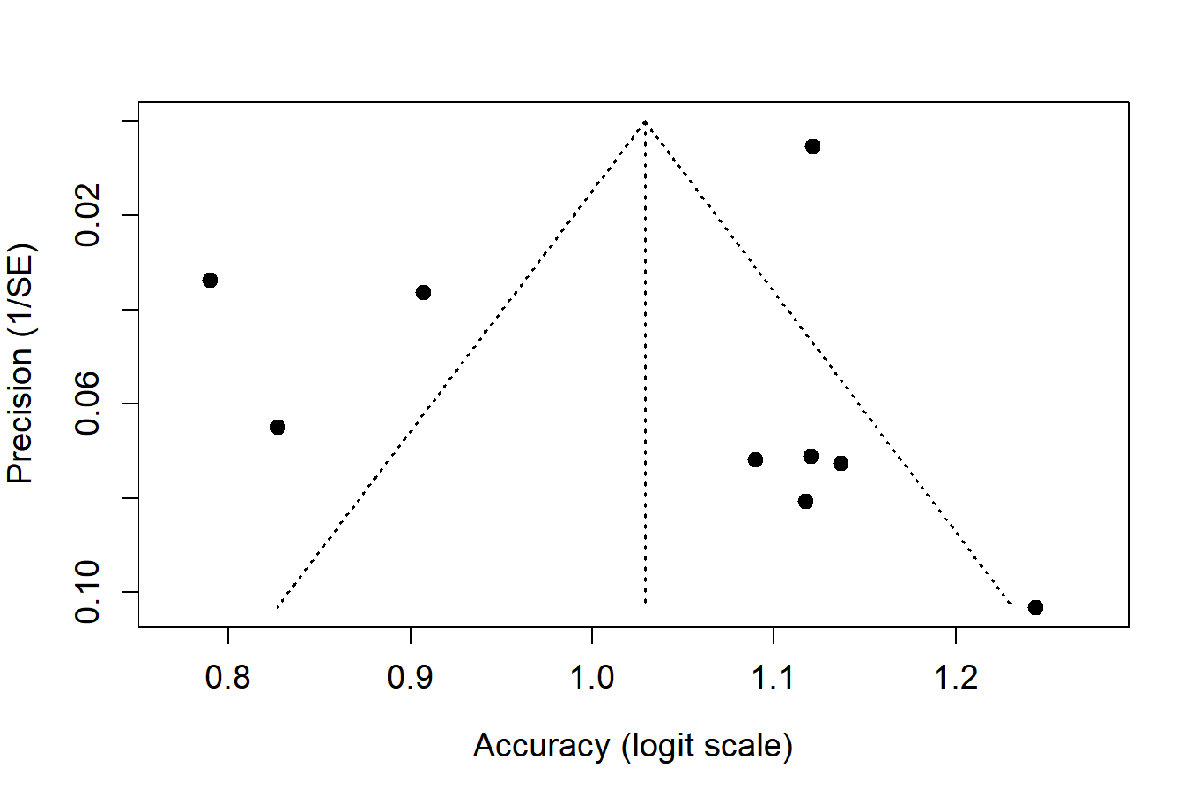

Supplement: Multimedia Appendix 1 [file jmir-v28-e86079-s001.docx]
