# Supplementary material for: Artificial Intelligence for Predicting Treatment Response in Patients With Anxiety Disorders After Cognitive Behavioral Therapy: Systematic Review and Meta-Analysis
Source: J Med Internet Res. 2026 Mar 18;28:e86079. doi: 10.2196/86079 (PMC12998711; doi:10.2196/86079)
Supplement: Multimedia Appendix 2 [file jmir-v28-e86079-s002.docx]

**Multimedia Appendix 2.** Technical aspects and diagnostic performance data extracted from the included studies.

| **Author** | **Year** | **Data splitting method** | **Type of anxiety disorders** | **Total patients (No. of response patients)** | **Predictors** | **AI algorithms** | **AI method** | **Internal validation datasets** | | | | |
| --- | --- | --- | --- | --- | --- | --- | --- | --- | --- | --- | --- | --- |
|  |  |  |  |  |  |  |  | **TP** | **FP** | **FN** | **TN** | **Accuracy** |
| Ball et al [1] | 2014 | OOB x 500 | Anxiety | 48(29) | Clinical and  demographic | RF | ML | 23 | 9 | 6 | 10 | 0.69(33/48) |
|  |  |  |  |  | Task fMRI |  |  | 25 | 6 | 4 | 13 | 0.79(38/48) |
|  |  |  |  |  | Multimodal |  |  | 24 | 8 | 5 | 11 | 0.73(35/48) |
|  |  |  | GAD | 22(13) | Clinical and  demographic |  |  | 10 | 2 | 3 | 7 | 0.77(17/22) |
|  |  |  |  |  | Task fMRI |  |  | 10 | 3 | 3 | 6 | 0.73(16/22) |
|  |  |  |  |  | Multimodal |  |  | 11 | 2 | 2 | 7 | 0.82(18/22) |
|  |  |  | PD | 20(14) | Clinical and  demographic |  |  | 13 | 1 | 1 | 5 | 0.90(18/20) |
|  |  |  |  |  | Task fMRI |  |  | 13 | 2 | 1 | 4 | 0.85(17/20) |
|  |  |  |  |  | Multimodal |  |  | 12 | 2 | 2 | 4 | 0.80(16/20) |
| Bertie et al [2] | 2024 | 10-fold cross-validation | GAD, Separation anxiety, Social anxiety, Specific phobias | 2214(NA) | Clinical and demographic | LR, LGBM, NODE | ML, DL | NA | NA | NA | NA | NA |
| Bukhari et al [3] | 2025 | Nested | SAD | 157(NA) | Clinical and demographic | Lasso,  Ridge regression SVR,  Extra trees | ML | NA | NA | NA | NA | NA |
| Frick et al [4] | 2020 | LOOCV | SAD | 47(24) | Clinical and  demographic | SVM | ML | 16 | 16 | 8 | 7 | 0.49(23/47) |
|  |  |  |  |  | Task fMRI |  |  | 21 | 5 | 3 | 18 | 0.83(39/47) |
|  |  |  |  |  | Multimodal |  |  | 18 | 9 | 6 | 14 | 0.68(32/47) |
| Hahn et al [5] | 2015 | Nested, LOOCV | PD with AG | 49(25) | Task fMRI | SVM | ML | 23 | 7 | 2 | 17 | 0.82(40/49) |
|  |  |  |  |  | Multimodal |  |  | 19 | 4 | 6 | 20 | 0.80(39/49) |
| Hilbert et al [6] | 2024 | Independent validation | PD, AG, SAD, SPH | Protect-AD  220(108) | Clinical and  demographic | RF | ML | 50 | 59 | 58 | 53 | 0.47(103/220) |
|  |  |  |  |  | rs-fMRI (Connectivity) | RF | ML | 58 | 59 | 50 | 53 | 0.50(111/220) |
|  |  |  |  |  | rs-fMRI (Graph Metrics) | RF | ML | 55 | 57 | 53 | 55 | 0.50(110/220) |
|  |  |  |  | SpiderVR:  190(105) | Clinical and  demographic | RF | ML | 72 | 39 | 33 | 46 | 0.62(118/190) |
|  |  |  |  |  | rs-fMRI  (SPQ prepost) | RF | ML | 60 | 41 | 45 | 44 | 0.55(104/190) |
|  |  |  |  |  | rs-fMRI  (SPQ preFU) | SVM | ML | 59 | 48 | 46 | 37 | 0.51(96/190) |
|  |  |  |  |  | Multimodal  (SPQ prepost) | SVM | ML | 65 | 40 | 40 | 45 | 0.58(110/190) |
|  |  |  |  |  | Multimodal  (SPQ preFU) | RF | ML | 61 | 53 | 44 | 32 | 0.49(93/190) |
| Isacsson et al [7] | 2024 | 10-fold cross-validation | Panic | 1767 | Multimodal | Lasso | ML | NA | NA | NA | NA | 0.72(1281/1767) |
|  |  |  |  |  |  | ElasticNet | ML | NA | NA | NA | NA | 0.75(1327/1767) |
|  |  |  |  |  |  | KNN | ML | NA | NA | NA | NA | 0.72(1274/1767) |
|  |  |  |  |  |  | GradientBoost | ML | NA | NA | NA | NA | 0.70(1230/1767) |
|  |  |  |  |  |  | Ada | ML | NA | NA | NA | NA | 0.76(1338/1767) |
|  |  |  |  |  |  | RF | ML | NA | NA | NA | NA | 0.76(1339/1767) |
|  |  |  |  |  |  | LinearSVR | ML | NA | NA | NA | NA | 0.75(1322/1767) |
|  |  |  |  |  |  | Ridge | ML | NA | NA | NA | NA | 0.74(1313/1767) |
|  |  |  |  |  |  | BayesianRidge | ML | NA | NA | NA | NA | 0.74(1308/1767) |
|  |  |  | Social Anxiety | 1852 |  | Lasso | ML | NA | NA | NA | NA | 0.73(1350/1852) |
|  |  |  |  |  |  | ElasticNet | ML | NA | NA | NA | NA | 0.74(1369/1852) |
|  |  |  |  |  |  | KNN | ML | NA | NA | NA | NA | 0.71(1313/1852) |
|  |  |  |  |  |  | GradientBoost | ML | NA | NA | NA | NA | 0.76(1402/1852) |
|  |  |  |  |  |  | Ada | ML | NA | NA | NA | NA | 0.76(1413/1852) |
|  |  |  |  |  |  | RF | ML | NA | NA | NA | NA | 0.77(1426/1852) |
|  |  |  |  |  |  | LinearSVR | ML | NA | NA | NA | NA | 0.76(1400/1852) |
|  |  |  |  |  |  | Ridge | ML | NA | NA | NA | NA | 0.76(1404/1852) |
|  |  |  |  |  |  | BayesianRidge | ML | NA | NA | NA | NA | 0.75(1393/1852) |
| Månsson et al [8] | 2015 | LOOCV | SAD | 23(12) | Task fMRI (ACC) | SVM | ML | 10 | 0 | 2 | 11 | 0.91(21/23) |
|  |  |  |  |  | Task fMRI (Amygdala) |  |  | 6 | 6 | 6 | 5 | 0.48(11/23) |
|  |  |  |  |  | Task fMRI (dIPFC) |  |  | 6 | 7 | 6 | 4 | 0.43(10/23) |
|  |  |  |  |  | Task fMRI (Hippocampus) |  |  | 7 | 6 | 5 | 5 | 0.52(12/23) |
|  |  |  |  |  | Task fMRI (Insula) |  |  | 5 | 6 | 7 | 5 | 0.43(10/23) |
|  |  |  |  |  | Task fMRI (vmPFC) |  |  | 5 | 7 | 7 | 4 | 0.39(9/23) |
| Prasad et al [9]^a^ | 2023 | Train/Validation/Test | Anxiety | 9240(3512) | Clinical and  demographic | RNN | DL | 2160 | 395 | 1352 | 5333 | 0.81(7493/9240) |
| Prasad et al [9]^b^ | 2023 | Train/Validation/Test | Anxiety | 4617(1720) | Clinical and  demographic | RNN | DL | 1003 | 174 | 717 | 2723 | 0.81(3726/4617) |
| Sundermann et al [10] | 2017 | LOOCV | PD with AG | 59(30) | Task fMRI-  Interoceptive attention (No FS) | SVM | ML | 11 | 17 | 19 | 12 | 0.39(23/59) |
|  |  |  |  |  | Task fMRI-  Interoceptive attention (FS t-test) |  |  | 12 | 18 | 18 | 11 | 0.39(23/59) |
|  |  |  |  |  | Task fMRI-Interoceptive attention (SVM-RFE) |  |  | 11 | 17 | 19 | 12 | 0.39(23/59) |
|  |  |  |  |  | Task fMRI-Interoceptive > exteroceptive (No FS) |  |  | 9 | 15 | 21 | 14 | 0.39(23/59) |
|  |  |  |  |  | Task fMRI-Interoceptive > exteroceptive (FS t-test) |  |  | 15 | 12 | 15 | 17 | 0.54(32/59) |
|  |  |  |  |  | Task fMRI-Interoceptive > exteroceptive (SVM-RFE) |  |  | 12 | 16 | 18 | 13 | 0.42(25/59) |
| Whitﬁeld-Gabrieli et al [11] | 2015 | LOOCV | SAD | 38(19) | Multimodal | LR | ML | 16 | 4 | 3 | 15 | 0.82(31/38) |

ML Machine learning; DL Deep learning; TP true positive; TN true negative; FP false positive; FN false negative; NA not available;  CBT cognitive behavioral therapy; GAD generalized anxiety disorder; PD panic disorder; SAD social anxiety disorder; SPH specific phobia; PD with AG panic disorder with agoraphobia; AG agoraphobia; RF random forest; LR logistic regression; LGBM light gradient boosting machine; NODE neural oblivious decision ensembles; SVR support vector regression;  SVM support vector machine; Lasso least absolute shrinkage and selection operator; KNN k-nearest neighbors; Ada adaboost regressor; RNN recurrent neural network; SVM-RFE  support vector machine - recursive feature elimination; ACC anterior cingulate cortex; dIPFC dorsolateral prefrontal cortex; vmPFC  ventromedial prefrontal cortex; FS feature selection; fMRI  functional magnetic resonance imaging; dMRI diffusion-weighted magnetic resonance imaging; OBB out-of-bag; LOOCV leave-One-Out Cross-Validation.

^a^ from validation sets in GAD-7 measures. ^b^ from test sets in GAD-7 measures.

**References**

1. Ball TM, Stein MB, Ramsawh HJ, Campbell-Sills L, Paulus MP. Single-subject anxiety treatment outcome prediction using functional neuroimaging. Neuropsychopharmacology. Apr 2014;39(5):1254-1261. [doi: 10.1038/npp.2013.328]

2. Bertie LA, Quiroz JC, Berkovsky S, et al. Predicting remission following CBT for childhood anxiety disorders: a machine learning approach. Psychol Med. Dec 17, 2024;17:1-11. [doi: 10.1017/S0033291724002654]

3. Bukhari Q, Rosenfield D, Hofmann SG, Gabrieli JDE, Ghosh SS. Predicting treatment response to cognitive behavior therapy in social anxiety disorder on the basis of demographics, psychiatric history, and scales: a machine learning approach. PLoS ONE. 2025;20(3):e0313351. [doi: 10.1371/journal.pone.0313351]

4. Frick A, Engman J, Alaie I, et al. Neuroimaging, genetic, clinical, and demographic predictors of treatment response in patients with social anxiety disorder. J Affect Disord. Jan 15, 2020;261:230-237. [doi: 10.1016/j.jad.2019.10.027]

5. Hahn T, Kircher T, Straube B, et al. Predicting treatment response to cognitive behavioral therapy in panic disorder with agoraphobia by integrating local neural information. JAMA Psychiatry. Jan 2015;72(1):68-74. [doi: 10.1001/jamapsychiatry.2014.1741]

6. Hilbert K, Böhnlein J, Meinke C, et al. Lack of evidence for predictive utility from resting state fMRI data for individual exposure-based cognitive behavioral therapy outcomes: a machine learning study in two large multi-site samples in anxiety disorders. Neuroimage. Jul 15, 2024;295(120639):120639. [doi: 10.1016/j.neuroimage.2024.120639]

7. Hentati Isacsson N, Ben Abdesslem F, Forsell E, Boman M, Kaldo V. Methodological choices and clinical usefulness for machine learning predictions of outcome in Internet-based cognitive behavioural therapy. Commun Med (Lond). Oct 10, 2024;4(1):196. [doi: 10.1038/s43856-024-00626-4]

8. Månsson KNT, Frick A, Boraxbekk CJ, et al. Predicting long-term outcome of Internet-delivered cognitive behavior therapy for social anxiety disorder using fMRI and support vector machine learning. Transl Psychiatry. Mar 17, 2015;5(3):e530. [doi: 10.1038/tp.2015.22]

9. Prasad N, Chien I, Regan T, et al. Deep learning for the prediction of clinical outcomes in internet-delivered CBT for depression and anxiety. PLoS ONE. 2023;18(11):e0272685. [doi: 10.1371/journal.pone.0272685]

10. Sundermann B, Bode J, Lueken U, et al. Support vector machine analysis of functional magnetic resonance imaging of interoception does not reliably predict individual outcomes of cognitive behavioral therapy in panic disorder with agoraphobia. Front Psychiatry. 2017;8:99. [doi: 10.3389/fpsyt.2017.00099]

11. Whitfield-Gabrieli S, Ghosh SS, Nieto-Castanon A, et al. Brain connectomics predict response to treatment in social anxiety disorder. Mol Psychiatry. May 2015;21(5):680-685. [doi: 10.1038/mp.2015.109]
